# Kinase Inhibitor Screening Identifies Cyclin-Dependent Kinases and Glycogen Synthase Kinase 3 as Potential Modulators of TDP-43 Cytosolic Accumulation during Cell Stress

**DOI:** 10.1371/journal.pone.0067433

**Published:** 2013-06-26

**Authors:** Diane Moujalled, Janine L. James, Sarah J. Parker, Grace E. Lidgerwood, Clare Duncan, Jodi Meyerowitz, Takashi Nonaka, Masato Hasegawa, Katja M. Kanninen, Alexandra Grubman, Jeffrey R. Liddell, Peter J. Crouch, Anthony R. White

**Affiliations:** 1 Department of Pathology, The University of Melbourne, Victoria, Australia and Florey Institute of Neuroscience and Mental Health, Parkville, Victoria, Australia; 2 Department of Neuropathology and Cell Biology, Tokyo Metropolitan Institute of Medical Science, Setagaya-ku, Tokyo, Japan; 3 A.I. Virtanen Institute for Molecular Sciences, Laboratory of Molecular Brain Research, University of Eastern Finland, Kuopio, Finland; International Centre for Genetic Engineering and Biotechnology, Italy

## Abstract

Abnormal processing of TAR DNA binding protein 43 (TDP-43) has been identified as a major factor in neuronal degeneration during amyotrophic lateral sclerosis (ALS) or frontotemporal lobar degeneration (FTLD). It is unclear how changes to TDP-43, including nuclear to cytosolic translocation and subsequent accumulation, are controlled in these diseases. TDP-43 is a member of the heterogeneous ribonucleoprotein (hnRNP) RNA binding protein family and is known to associate with cytosolic RNA stress granule proteins in ALS and FTLD. hnRNP trafficking and accumulation is controlled by the action of specific kinases including members of the mitogen-activated protein kinase (MAPK) pathway. However, little is known about how kinase pathways control TDP-43 movement and accumulation. In this study, we used an *in vitro* model of TDP-43-positve stress granule formation to screen for the effect of kinase inhibitors on TDP-43 accumulation. We found that while a number of kinase inhibitors, particularly of the MAPK pathways modulated both TDP-43 and the global stress granule marker, human antigen R (HuR), multiple inhibitors were more specific to TDP-43 accumulation, including inhibitors of cyclin-dependent kinases (CDKs) and glycogen synthase kinase 3 (GSK3). Close correlation was observed between effects of these inhibitors on TDP-43, hnRNP K and TIAR, but often with different effects on HuR accumulation. This may indicate a potential interaction between TDP-43, hnRNP K and TIAR. CDK inhibitors were also found to reverse pre-formed TDP-43-positive stress granules and both CDK and GSK3 inhibitors abrogated the accumulation of C-terminal TDP-43 (219–414) in transfected cells. Further studies are required to confirm the specific kinases involved and whether their action is through phosphorylation of the TDP-43 binding partner hnRNP K. This knowledge provides a valuable insight into the mechanisms controlling abnormal cytoplasmic TDP-43 accumulation and may herald new opportunities for kinase modulation-based therapeutic intervention in ALS and FTLD.

## Introduction

Amyotrophic lateral sclerosis (ALS) is a fatal adult-onset motor neuron disease that commonly strikes people between 40 and 60 years of age. During the disease, motor neurons in the spinal cord and brain degenerate, normally leading to death in 1–5 years. The progressive deterioration of patients with ALS results in enormous healthcare and social costs, yet little is known about the disease process and no long-term effective treatments exist. Frontotemporal lobar degeneration (FTLD) is a collective term for a group of neurodegenerative diseases associated with degeneration in the frontal and temporal lobes of the brain [1]. FTLD is one of the most common causes of age-related dementia and while the symptoms of ALS and FTLD are generally distinct, some overlap has been reported [2].

The majority of ALS cases are sporadic but ∼5% of patients have a familial mutation. In 2006, TAR DNA binding protein 43 (TDP-43) was identified as a major protein constituent within ubiquitinated neuronal inclusions in a large proportion of ALS and FTLD cases [3], [4]. This has led to the re-classification of many ALS and FTLD-ubiquitin cases as primary TDP-43 proteinopathies. TDP-43 has reported roles in RNA processing including transcription, pre-mRNA splicing, and transport and stabilization of mRNA [2]. Although the majority of TDP-43 is normally localized to the cell nucleus, the protein can shuttle between the nucleus and cytosol [5]. However, in TDP-43 proteinopathies there is substantial clearance of nuclear TDP-43 together with accumulation of ubiquitinated and hyperphosphorylated C-terminal fragment (CTF-TDP-43) in cytoplasmic inclusions [2], [3]. Recapitulation of these effects in cells transfected with CTF-TDP-43 supports a role for cytosolic TDP-43 accumulation in subsequent neuronal cell death [6]. However, little is known about the mechanisms that control translocation of TDP-43 to the cytosol or how TDP-43 becomes accumulated in these diseases.

TDP-43 has been found to associate with cytosolic RNA stress granule (SG) proteins. This may be an essential early step in pathological accumulation of TDP-43 [7], [8]. Cell-lines transfected with mutant or CTF-TDP-43 reveal association of cytosolic TDP-43 with various SG proteins including, T-cell intracytoplasmic antigen (TIA-1), human antigen R (HuR) and additional hnRNPs such as hnRNP A1, A3 and K [7]–[9]. In addition, SG proteins have been co-localized with cytosolic TDP-43 inclusions in ALS spinal cord and FTLD brain tissue [8]. Another RNA-binding protein found to cause ALS i.e. ‘fused in sarcoma’ (FUS) also associates with SG proteins in transfected cells and human disease tissue [9]–[12]. RNA SGs are sites of stalled mRNA pre-initiation complexes. During stress, cells stall mRNA translation of non-critical proteins to transfer energy expenditure to translation of key survival proteins. After cessation of stress, SGs normally dissociate to allow mRNA processing to continue. It is not known how TDP-43 becomes associated with SG proteins in cell models or *in vivo* and how this may contribute to the disease process.

It is well known that protein kinases control movement and accumulation of SG proteins including hnRNPs and HuR [13]–[18]. Mitogen-activated protein kinases (MAPK), which include c-Jun N-terminal kinase (JNK), p38 and extracellular signal regulated kinase (ERK), control translocation of hnRNPs from the nucleus to cytosol and subsequent accumulation into SGs [19]. JNK-mediated phosphorylation of hnRNP K at Ser216/Ser353 results in cytoplasmic accumulation [16] and JNK modulates localization and activity of additional SG proteins [18]. p38 has been reported to control cytoplasmic accumulation of hnRNP A1 during osmotic shock or senescence [17], [20], [21] and HuR interactions with mRNA during anisomycin treatment [22]. ERK modulates movement of hnRNP K within carcinoma cells [19] and in response to T-cell activation [13]. Recently, we demonstrated that JNK specifically controlled localization of TDP-43 to SGs induced by mitochondrial inhibition and had a partial role in modulating accumulation of CTF-TDP-43 in transfected cells [23], [24]. We have also shown that inhibition of ERK by treatment of cells with copper-based metallo-complexes can prevent TDP-43 and HuR cytosolic accumulation via modulation of processes associated with ubiquitination [25]. Other studies have shown that there are potential additional interactions between kinases and TDP-43. Ayala et al., demonstrated the co-localization of TDP-43 and ERK within inclusions of ALS patients [26].

Whether additional kinases have a critical role in controlling TDP-43 nuclear to cytosolic trafficking and subsequent accumulation in the cytosol is not clear. Glycogen synthase kinase 3 (GSK3) has a central role in neurodegeneration due to its modulation of the microtubule-associated protein, tau [27], [28]. GSK3 occurs in TDP-43-positive aggregates in cells and possibly *in vivo* [29] and controls cytosolic trafficking of hnRNPs [30]. Furthermore, cyclin-dependent kinases (CDKs) have also been reported to control the subcellular trafficking of SG proteins and protein aggregation in neurodegenerative diseases [31]–[34]. These kinases (JNK, GSK3 and CDKs) have all been linked to neuronal cell dysfunction in ALS and FTLD [35]–[39] but their role in TDP-43 trafficking is not known.

In the present study we examined whether additional kinases are involved in accumulation of TDP-43 in SGs in cell culture. Using our established model of mitochondrial inhibition to induce TDP-43-positive SGs in SH-SY5Y cells, we screened 80 kinase inhibitors covering 35 different kinases (Tocriscreen kinase inhibitor toolbox) to determine the effect of kinase inhibition on TDP-43 localization to SGs.

## Methods

### Materials

4′,6′ Diamino-2-phenylindole dihydrochloride (DAPI) was obtained from Invitrogen (Mount Waverley, Victoria, Australia). N,N′-dimethyl-4,4′-bipyridinium dichloride (paraquat) and sodium arsenite, were from Sigma Aldrich (Sydney, NSW, Australia). Tocriscreen *kinase inhibitor toolbox* was from Tocris Bioscience (Ellisville, Melbourne, Victoria, Australia).

Polyclonal TDP-43 antisera were purchased from Proteintech Group (Chicago, IL, USA). Polyclonal antisera to hnRNP K were purchased from Abcam (Waterloo, Australia). Monoclonal antisera to hnRNP A1 were from Merck (Kilsyth, Victoria, Australia). TIA-1-related (TIAR) polyclonal antisera were from Cell Signaling Technology (Arundal, Queensland, Australia). Monoclonal antisera to HuR were obtained from Invitrogen (Mount Waverley, Victoria, Australia).

### Cell Culture

The cell lines used in this study were human neuroblastoma SH-SY5Y cell line and the human epithelial HeLa cell line. Cells were passaged and maintained in DMEM plus 5% FBS (HeLa cells) or DMEM/F12 plus 10% FBS (SH-SY5Y cells). To induce differentiation, SH-SY5Y cells were treated with 10 µM retinoic acid for 7 days. Differentiation was confirmed by morphological changes (neurite extension) and up-regulated expression of synaptophysin, tyrosine hydroxylase and VMAT2 [23]. All cells were grown in 5% CO_2_ at 37°C.

### Exposure of Cell to Stress

Undifferentiated cells were grown in 24 or 6-well plates or on 12 mm coverslips (for immunofluorescence) for 2–3 days before experiments (∼80% confluent). Where indicated, retinoic acid-treated SH-SY5Y cells were cultured for 7 days before experiments. Paraquat or sodium arsenite was prepared in dH_2_O and added at indicated concentrations and the medium was briefly mixed by aspiration. Incubations were performed for periods stated in individual experiments. Where indicated, cells were co-treated with kinase inhibitors at 10 µM from Tocriscreen kinase inhibitor toolbox stock solutions prepared at 10 mM in DMSO. See Table S1 for list of abbreviations and full names of kinase targets. Where inhibitors induced cellular toxicity as determined by MTT assay [23], 1 µM was used. If 1 µM was found to be toxic, then further experiments were not attempted. Control cultures were treated with vehicle alone. For immunoblotting, cells were harvested into Phosphosafe Extraction Buffer (Merck Biosciences, San Diego, CA, USA) containing protease inhibitor cocktail (Roche Diagnostics, Hawthorn, Victoria, Australia) and stored at -80°C until use. For immunofluorescence studies, cells were grown on glass coverslips and fixed by treating with 4% paraformaldehyde for 30 min.

### Western Blot Analysis of Protein Expression and Phosphorylation

Cell lysates prepared in Phosphosafe Extraction Buffer at equal protein concentration were mixed with electrophoresis SDS sample buffer and separated on 12% SDS-PAGE Tris-Glycine gels. Proteins were transferred to PVDF membranes and blocked with 4% skim milk solution in PBST before immunoblotting. For detection of total TDP-43, membranes were probed with polyclonal antisera (1∶1,500) against TDP-43. For detection of total and phospho-forms of extracellular signal regulated protein (ERK), CDK2 and p38, polyclonal antisera from Cell Signaling Technology was used at 1∶5,000. Secondary antiserum was rabbit-HRP at 1∶5,000 dilution. Blots were developed using GE Healthcare ECL Advance Chemiluminescence (Rydelmere, NSW, Australia) and imaged on a Fujifilm LAS3000 imager (Berthold, Bundoora, Victoria, Australia). Expression of GAPDH was determined using antisera at 1∶5,000 for protein loading controls where necessary. Densitometric analysis of TDP-43 expression was performed using NIH Image J quantification of protein bands from at least three separate cultures.

### Immunofluorescence Analysis

SH-SY5Y cells, or HeLa cells, were grown on 12 mm diameter coverslips and treated with stress inducers and kinase inhibitors as indicated. Cells were fixed with 4% w/v paraformaldehyde in PBS for 30 min and permeabilized with 90% chilled methanol for 5 min. After blocking for 1 h with 10% normal goat serum, cells were incubated with primary antibody for total TDP-43 (1∶1,500), HuR (1∶50), TIAR (1∶40), hnRNP A1 (1∶200) or hnRNP K (1∶200) for 2 h at room temperature or overnight at 4°C. This was followed by labeling with secondary AlexaFluor or FITC goat anti-mouse or anti-rabbit antisera at 1∶500 for 2 h at room temperature or overnight at 4°C. After washing, the coverslips were incubated with DAPI at 0.5 µg/ml for 5 min and analyzed using a Leica inverted microscope with Zeiss Axiocam digital camera. Coverslips were examined blinded to the inhibitor treatment and the numbers of TDP-43, HuR, hnRNP K or TIAR-positive stress granules were manually counted in pre-determined multiple fields of view of two to three coverslips for each treatment. Where inhibitors increased or decreased stress granules, treatments were repeated twice. Stress granules were determined as positive if they were distinctly brighter than the surrounding cytoplasmic or nuclear immunofluorescence and/or were clearly separated from surrounding immunofluorescence to allow positive identification of a discrete structure. Results are presented as a percentage change compared to the number of stress-granule-positive cells from paraquat treatment alone. Images shown are representative of multiple fields and replicate or triplicate coverslips per experiment.

### Preparation of TDP-43 Plasmids

Plasmid DNA corresponding to GFP-tagged full-length wild-type (WT) TDP-43 (pEGFP-TDP WT) or C-terminal fragments of TDP-43, (pEGFP-TDP 219–414) or empty expression vector pEGFP-C1 were prepared as described by Nonaka et. al. [40]. Briefly, plasmid DNA was used to transform MAX Efficiency® DH5α™ Competent Cells (Invitrogen, Mount Waverley, Victoria, Australia) as described by the manufacturer. Transformants were grown and colonies were picked based on kanamycin-resistance and grown in liquid culture for subsequent plasmid purification. DNA was purified using the Wizard® *Plus* Midiprep DNA Purification System (Promega Corporation) as per manufacturer’s instructions. DNA was quantified and TDP-43 inserts were identified positively by digestion with *BamHI* and *XhoI.*

### Transfection and Expression of Plasmids

SH-SY5Y cells were seeded at 2×10^5^ cells per well in 24 well–plates on coverslips. Non-retinoic acid-treated cells were transfected 24 h after seeding with the pEGFP-C1 empty vector, pEGFP-TDP WT, and pEGFP-TDP 219–414 using Attractene (Qiagen) according to manufacturer’s instructions. Kinase inhibitors were added at 10 or 1 µM after 24 h. After a further 24 h incubation, cells were fixed with 4% w/v paraformaldehyde in PBS for 30 min. and permeabilized with 90% chilled methanol for 5 min. After washing, the coverslips were incubated with DAPI at 0.5 µg/ml for 5 min and analyzed using a Leica inverted microscope with Zeiss Axiocam digital camera. Expression of TDP-43 was determined by the EGFP-tagged construct [23].

### Statistical Analysis

All data described in graphical representations are mean ± standard error of the mean (SEM) unless stated. Results were analysed using a two-way ANOVA and Dunnett post-hoc test.

## Results

### Identification of Kinase Inhibitors that Modulate TDP-43 and/or HuR-positive Stress Granule Formation

**Figure 1:**
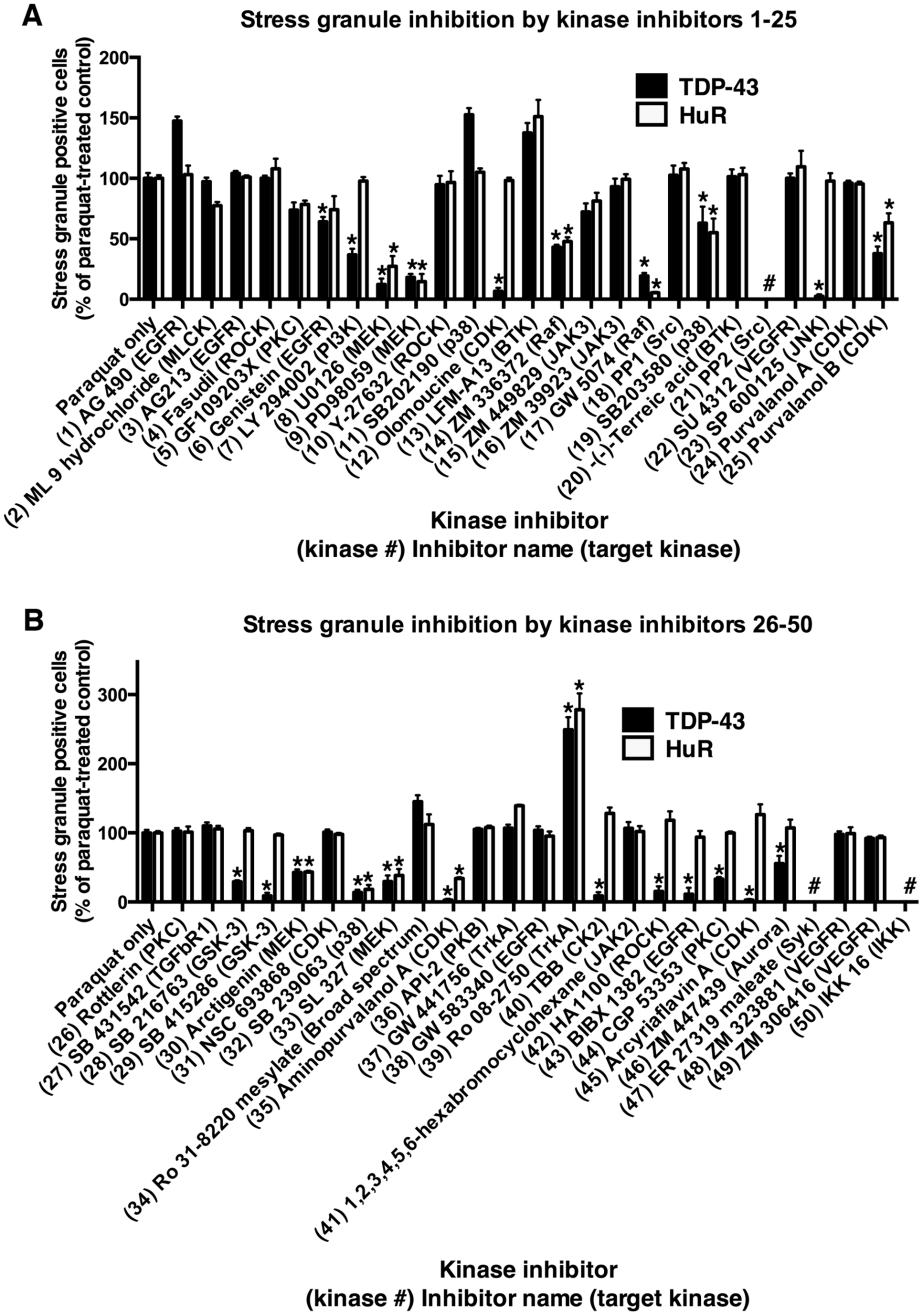
Stress granule inhibition by kinase inhibitors. SH-SY5Y cells were treated with 1 mM paraquat overnight in the presence or absence of kinase inhibitors 1–25 (**A**) and 26–50 (**B**) from the Tocriscreen kinase inhibitor toolbox. The numbers of stress granules positive for TDP-43 and/or HuR were counted and compared to cultures treated with paraquat treatment alone. *P<0.05 compared to paraquat treatment alone. # on graph indicates ‘not done’.

**Figure 2:**
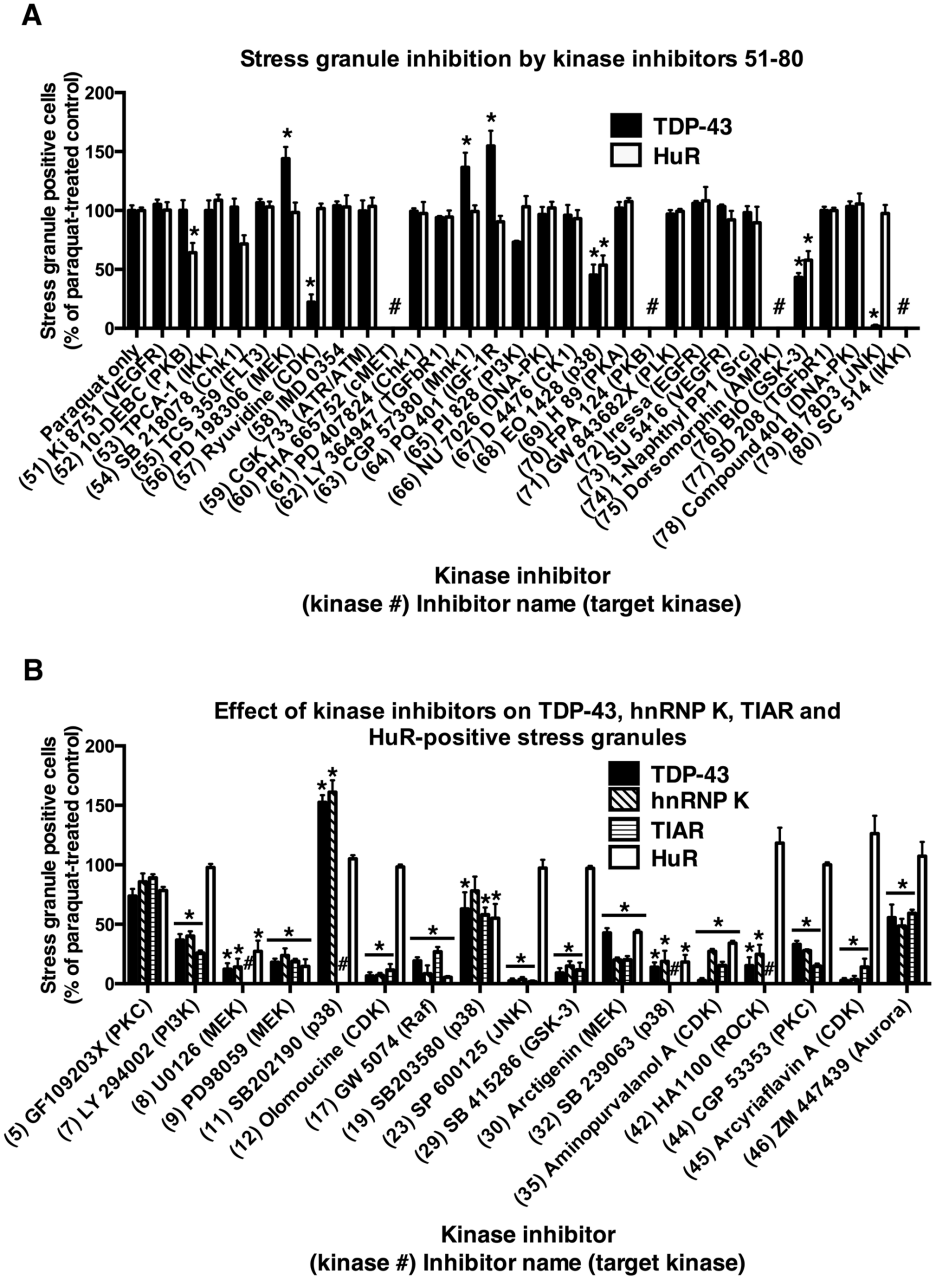
Stress granule inhibition by kinase inhibitors. SH-SY5Y cells were treated with 1 mM paraquat overnight in the presence or absence of kinase inhibitors 51–80 (**A**) or selected inhibitors (**B**) from the Tocriscreen kinase inhibitor toolbox. The numbers of stress granules positive for TDP-43 and/or HuR (**A**) or TDP-43, HnRNP K, TIAR and/or HuR (**B**) were counted and compared to cultures treated with paraquat treatment alone. *P<0.05 compared to paraquat treatment alone. # on graph indicates ‘not done’.

To examine the role of different kinase pathways in stress-induced accumulation of TDP-43, we exposed non-differentiated SH-SY5Y neurons to 1 mM paraquat overnight in the presence or absence of 80 different kinases inhibitors covering 35 separate kinases (Tocriscreen kinase inhibitor toolbox). Each inhibitor was used at a concentration of 10 µM, except where indicated, and coverslips were examined for the number of cells containing TDP-43- and/or HuR-positive stress granules. Paraquat-treated cells revealed robust formation of TDP-43 and/or HuR-positive stress cytosolic granules consistent with our previous studies (Figure S1 and [23], [24]. As reported previously, some cells also displayed diffuse cytosolic TDP-43 (Figure S1). Stress granules were rarely observed in untreated cultures. Upon co-treatment with kinase inhibitors, a range of effects were observed on TDP-43 and/or HuR-positive stress granule formation (see Figures 1 and 2, Table S2 and representative photomicrographs in Figures S1, S2, S3). Treatment with PD98059 (ERK inhibitor, #9), SB 203580 (p38 inhibitor, #19) SP600125 (JNK inhibitor, #23) or BI 78D3 (JNK inhibitor, #79) resulted in similar changes to TDP-43 and/or HuR stress granule formation as reported previously [23], [24]. This included a significant reduction in TDP-43 and HuR-positive stress granules by PD98059 and SB 203580 and reduction in TDP-43-positive stress granules but with little effect on HuR-positive stress granules by SP600125 (Figures 1 and 2 and Table S2).

Subsequently, we examined the effects of additional kinase inhibitors. The phosphoinositol-3-kinase (PI3K) inhibitor, LY294002 (#7) significantly reduced TDP-43-positive stress granule formation with no significant change to HuR-positive stress granules (Figure 1, Table S2 and representative image in Figure S1). Inhibitors of alternative kinases also induced a similar effect of inhibiting TDP-43-positive stress granule formation with little effect on HuR-positive stress granules. As shown in Figures 1 and 2 and Table S2, these included the CDK inhibitor, olomoucine (#12) (see representative image in Figure S1); GSK3 inhibitors, SB 216763 (#28) and SB 415286 (#29) (see representative image in Figure S1); casein kinase 2 (CK2) inhibitor, TBB (#40); ROCK inhibitor, HA 1100 hydrochloride (#42) (see representative image in Figure S3); epidermal growth factor inhibitor (EGFR), BIBX 1382 dihydrochloride (#43); protein kinase C (PKC) inhibitor, CGP 53353 (#44) (see representative image in Figure S3); CDK inhibitor, arcyriaflavin A (#45) (see representative image in Figure S3); aurora kinase inhibitor, ZM 447439 (#46) (see representative image in Figure S3); and CDK inhibitor, ryuvidine (#57).

Many additional kinase inhibitors either decreased or increased the number of both TDP-43 and HuR-positive stress granules. For example the MEK inhibitor, U0126 (#8) (Figure 1 and representative image in Figure S1); Raf inhibitors, ZM 336372 (#14) and GW 5074 (#17); p38 inhibitor SB 239063 (#32) (see representative image in Figure S1); GSK3 inhibitor BIO (#76) and several others as shown in Figures 1 and 2 and Table S2. Rarely, inhibitors decreased HuR-positive stress granule formation with no effect on TDP-43-positive stress granules (protein kinase B (PKB) inhibitor, 10-DEBC hydrochloride (#52) (Table S2). Interestingly, some inhibitors increased TDP-43 and/or HuR-positive stress granules (TrkA inhibitor, Ro 08-2750 (#39); MEK inhibitor, PD 198306 (#56); Mnk1 inhibitor, CGP 57380 (#63); and IGF-1R inhibitor, PQ 401 (#64) Figures 1 and 2).

### Multiple Inhibitors of CDK, GSK3 and MEK Pathways Inhibit TDP-43-positive Stress Granule Formation

From this screen, we identified that multiple inhibitors of particular kinases or known kinase pathways induced consistent effects on TDP-43-positive stress granule formation. As shown in Table S3 three of the four p38 inhibitors examined reduced formation of both TDP-43 and HuR-positive stress granules. Seven inhibitors of CDKs were examined, and of these, three reduced TDP-43-positive stress granule formation without effect on HuR (#12, #45 and #57). Two additional CDK inhibitors reduced TDP-43 and HuR-positive stress granule formation (#25 and #35). Only two had no effect on stress granule formation (#24 and #31). Additionally, as the aurora kinase inhibitor ZM 447439 (#46) reduced TDP-43 stress granule formation without effect on HuR-positive stress granules, we examined additional aurora kinase inhibitors 4-(4′-benzamidoanilino)-6,7-dimethoxyquinazoline and cyclopropanecarboxylic acid-(3-(4-(3-trifluoromethyl-phenylamino)-pyrimidin-2-ylamino)-phenyl)-amide, however, neither reduced TDP-43-positive stress granule formation. Aurora kinases are often associated with the same cellular processes as CDKs [41], therefore, it is possible that its effect on TDP-43-positive stress granules is also via a similar mechanism (Table S3). Similarly, four of five inhibitors against MEK reduced both TDP-43 and HuR-positive stress granule formation (Table S3). Three inhibitors of GSK3 were examined and two of these (#28 and #29) reduced TDP-43-positive stress granule formation with no effect on HuR while inhibitor #76 (BIO) reduced formation of both TDP-43 and HuR-positive stress granules (Table S3).

To determine if the action of some key kinase inhibitors reflected the ability of the inhibitor to abrogate phosphorylation of its target kinase, we performed a dose-response analysis on TDP-43-positive stress granule accumulation compared to kinase phosphorylation. As shown in Table S4, the inhibitors U0126 (#8, MEK) and olomoucine (#12, CDK) induced a dose-dependent inhibition of target kinase phosphorylation (phospho-ERK and phospho-CDK2 respectively). Alternatively, SB 203580 (#19, p38) did not show a dose-dependent action, suggesting that for some inhibitors, off-target effects could account for the inhibitory action on TDP-43 accumulation.

Inhibitors of some kinases showed a great deal of variation. Six inhibitors of EGFR were examined with a range of effects including no inhibition (#1, #3 #38 and #72), and inhibition of TDP-43-positive stress granules only (#6 and #43) (Table S3). Summarizing the data for Figures 1 and 2A and Table S3, clear and consistent changes to TDP-43-stress granule accumulation were observed using multiple inhibitors of p38, CDKs, GSK3 and MEK. It is also possible that alternative concentrations of inhibitors could produce different effects on both TDP-43 and HuR stress granule formation, however, broad dose testing of 80 inhibitors was not feasible in this model system.

### Inhibition of TDP-43-positive Stress Granule Formation was not Directly Attributed to Loss of TDP-43 Expression

**Figure 3:**
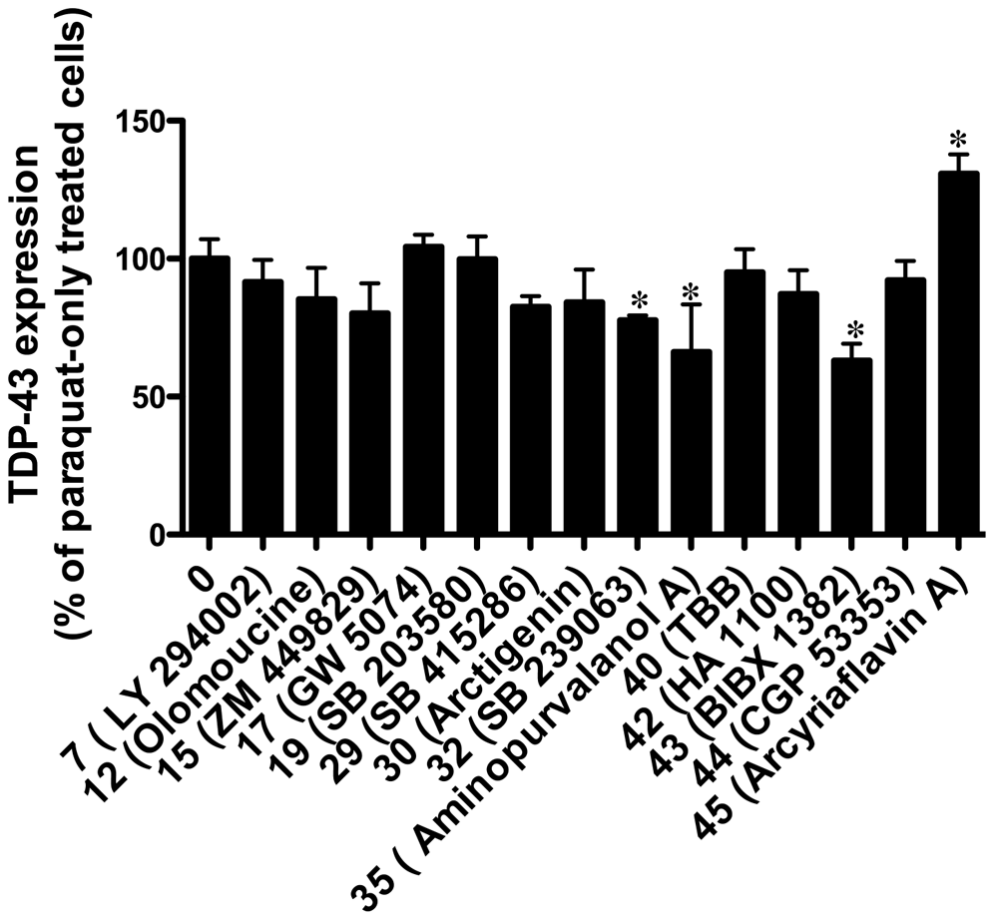
Effect of selected kinase inhibitors on TDP-43 expression. SH-SY5Y cells were treated with paraquat overnight in the presence or absence of 10 µM LY294002 (#7, PI3K); olomoucine (#12, CDKs); ZM 449829 (#15, JAK3); GW 5074 (#17, Raf); SB 203580 (#19, p38); SB 415286 (#29, GSK3); arctigenin (#30, MEK); SB 239063 (#32, p38); (1 µM) aminopurvalanol A (#35, CDKs); TBB (#40, CK2); HA 1100 (#42, ROCK); BIBX 1382 (#43, EGFR); CGP 53353 (#44, PKC); arcyriaflavin A (#45, CDKs). Western blot analysis of TDP-43 expression was determined and represented as densitometric analysis of expression compared to untreated control. *P<0.05 compared to untreated control.

The inhibition of TDP-43 stress granule formation by various kinase inhibitors could potentially reflect an inhibition of TDP-43 expression rather than inhibition of TDP-43 cytosolic accumulation. To investigate this, we measured the effect of selected inhibitors on TDP-43 expression in SH-SY5Y cells by western blot. Figure 3 and Figure S4 shows that inhibitors #32 (SB 239063, p38), #35 (aminopurvalanol A, CDK) and #43 (BIBX 1382 dihydrochloride, EGFR) induced a significant decrease in expression of TDP-43. However, the loss of TDP-43 expression did not directly match the large inhibition of TDP-43-positive stress granule formation observed in cultures treated with these inhibitors (Figure 1B). These results show that the effects of the inhibitors on TDP-43-positive stress granule formation were not simply due to loss of TDP-43 expression. However, given that the formation of stress granules may not be directly related to linear loss of protein, it remains a possibility that a reduced TDP-43 expression could affect the numbers of subsequent stress granules.

### Close Correlation between Inhibition of TDP-43, hnRNP K and TIAR-positive Stress Granules

Our initial broad kinase inhibitor screen (Figure 1 and Figure 2A) identified several kinases involved in modulation of TDP-43 accumulation in paraquat-treated cells (p38, CDKs, GSK3 and MEK). However, TDP-43 does not contain known phosphorylation consensus sites for these kinases or kinase pathways (i.e., MEK controls ERK, however, there are also no consensus sites for ERK phosphorylation on TDP-43). We reported previously that JNK specifically inhibited TDP-43-positive stress granule formation [23] and as TDP-43 does not contain a known JNK phosphorylation consensus site, the control of TDP-43 may have been through interaction with additional hnRNP or stress granule proteins. In support of this we showed that TDP-43 co-localized with hnRNP K, which is known to have multiple phosphorylation consensus sites and its accumulation is controlled by kinases [23]. Therefore, in this study, we compared the effect of selected kinase inhibitors on stress granule proteins known to interact with TDP-43, including hnRNP K and TIAR. There was close concordance between the inhibitory effect of selected kinase inhibitors on TDP-43, hnRNP K and TIAR-positive stress granule formation in paraquat-treated SH-SY5Y cells. Most inhibitors that abrogated TDP-43 accumulation into stress granules had an analogous effect on hnRNP K (for example, inhibitors LY294002 (#7, PI3K); U0126 (#8, MEK); olomoucine (#12, CDK); SB 415286 (#29, GSK3); and SB 239063 (#32, p38), Figure S5). Analogous effects were also found for most inhibitors when examined for inhibition of TIAR and TDP-43 (for example, LY294002 (#7, PI3K); olomoucine (#12, CDK); and SB 415286 (#29, GSK3), Figure S6). AS shown in Figure 2B and Table S5, LY294002 (#7, PI3K); olomoucine (#12, CDK); SP600125 (#23, JNK); SB 415286 (#29, GSK3); CGP 53353, #44, PKC; and arcyriaflavin A (#45, CDK) all revealed substantial inhibition of TDP-43, hnRNP K and TIAR with little effect on HuR-positive stress granules. These findings are consistent with a role for kinase control of TDP-43 through interaction of the protein with hnRNP K and/or TIAR. hnRNP K has phosphorylation consensus sites for JNK and CDKs but not GSK3. Additional kinase inhibitors affected all stress granule markers, including TDP-43, hnRNP K, TIAR (where examined) and HuR (U0129, #8, MEK; PD98059, #9, MEK; GW 5074, #17, Raf; and aminopurvalanol A, #35, CDK, Table S5). Summarizing these findings (Table S6), inhibition of CDKs, JNK or GSK3 often resulted in loss of TDP-43, hnRNP K and TIAR-positive stress granules with no change to HuR-positive stress granules. This is consistent with an important interaction between TDP-43, hnRNP K and TIAR while association of these proteins with HuR appears to be less stringent. We also observed that the CDK inhibitors, olomoucine (#12) and arcyriaflavin A (#45) did not inhibit paraquat-mediated hnRNP A1 accumulation in stress granules (Figure S7). These findings supported a CDK-related interaction between TDP-43, hnRNP K and TIAR but not HuR or hnRNP A1. In contrast, inhibition of p38 and MEK generally resulted in loss of accumulation of all markers examined suggesting global control of stress granules by these kinase pathways during stress from the mitochondrial inhibitor, paraquat.

### CDK, GSK3 and Additional Selected Kinase Inhibitors Prevented TDP-43-Positive Stress Granule Formation in Alternative Cell Models of Stress Induction

**Figure 4:**
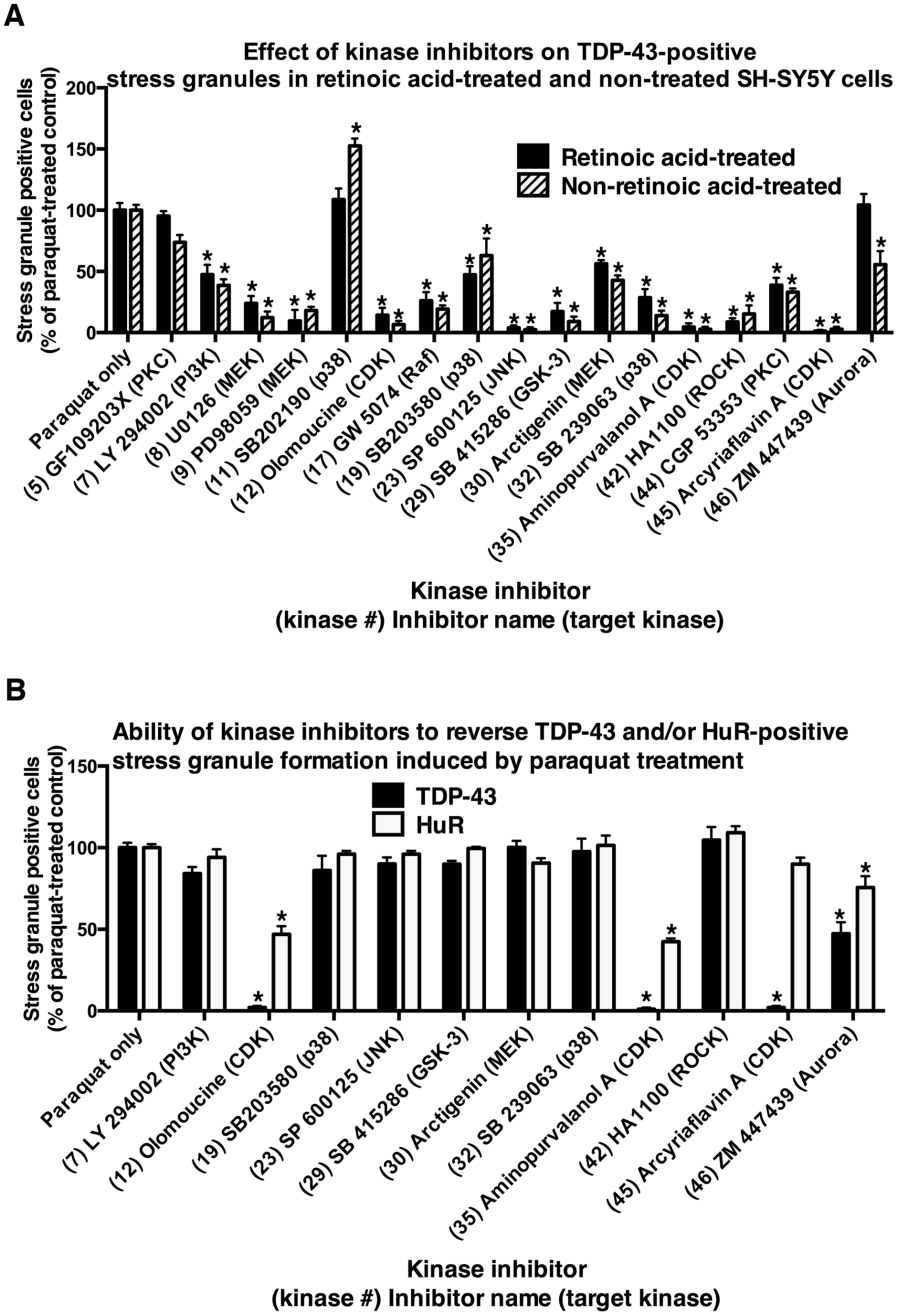
Control of stress granule formation in retinoic acid-treated SH-SY5Y cells, and reversal of stress granule formation in non retinoic acid-treated cells by selected kinase inhibitors. A: Stress granule inhibition by kinase inhibitors in retinoic acid-treated and non-treated SH-SY5Y cells. SH-SY5Y cells were treated with retinoic acid or left un-treated as described in Methods. Cells were then treated overnight with paraquat in the presence or absence of selected kinase inhibitors. The numbers of stress granules positive for TDP-43 were counted and compared to cultures with paraquat treatment alone. *P<0.05 compared to paraquat treatment alone. B: Ability of kinase inhibitors to reverse stress granule formation. Non-retinoic acid-treated SH-SY5Y cells were treated with paraquat overnight followed by 6 hr treatment in the presence or absence of selected kinase inhibitors. The numbers of stress granules positive for TDP-43 and/or HuR were counted and compared to cells treated with paraquat alone. *P<0.05 compared to paraquat treatment alone.

Next we examined if selected inhibitors identified in our initial screen of SH-SY5Y cells treated with paraquat induced similar effects on TDP-43 in additional models of stress. This was performed to determine if the effects of the inhibitors were consistent in alternate forms of stress. Initially we examined the effect of these inhibitors in retinoic acid-treated neurons [23]. As described previously, retinoic acid treatment induced a differentiated neuronal-like phenotype involving increased synaptophysin, tyrosine hydroxylase and VMAT2 [23]. This was performed to ensure that the effect of the CDK inhibitors was not related to cell cycle associated events in replicating cells. Figure 4 and Table S7 shows that of 17 kinase inhibitors tested in both non-treated and retinoic-acid treated SH-SY5Y cells, 14 revealed inhibition of TDP-43-positive stress granules in both. Importantly, CDK inhibitors olomoucine (#12) (see representative images in Figure S8), aminopurvalanol A (#35) and arcyriaflavin A (#45) inhibited TDP-43 stress granule accumulation in both models. We also treated HeLa cells with sodium arsenite to induce stress granule formation in this cell type. This treatment regime was used to examine if an alternative stress inducer in an un-related cell-type produces analogous effects with CDK inhibitors. The CDK inhibitors were found to inhibit TDP-43-positive stress granules from accumulating in HeLa epithelial cells exposed overnight to 50 µM sodium arsenite (Table S8 and see representative images in Figure S9).

### CDK Inhibitors can Reverse the Accumulation of Pre-formed TDP-43-positive Stress Granules

We previously reported that while JNK and ERK inhibitors were able to block formation of TDP-43-positive stress granules when added at the time of insult [23], addition of these inhibitors after formation of the TDP-43-positive stress granules failed to reverse the accumulation of the protein [24]. Therefore, in this study, we further examined the potential of selected inhibitors to reverse the formation of TDP-43-positive stress granules. Non-differentiated SH-SY5Y cells were treated overnight with 1 mM paraquat and the inhibitors were added for the final 6 h of incubation. We found that the CDK inhibitors, olomoucine (#12), aminopurvalanol A (#35) and arcyriaflavin A (#45) substantially reversed TDP-43-positive stress granule formation when added for the final 6 h (Figure 4B, Table S9 and see representative images in Figure S10). These findings indicate that while many kinase inhibitors can prevent the initial formation of TDP-43- (and HuR)- positive stress granules induced under the prolonged stress induction of paraquat, CDK inhibitors were able to successfully reverse the pre-accumulated TDP-43 positive stress granules.

### CDK, GSK3 and Additional Selected Kinase Inhibitors Inhibit Accumulation of C-terminal TDP-43

**Figure 5:**
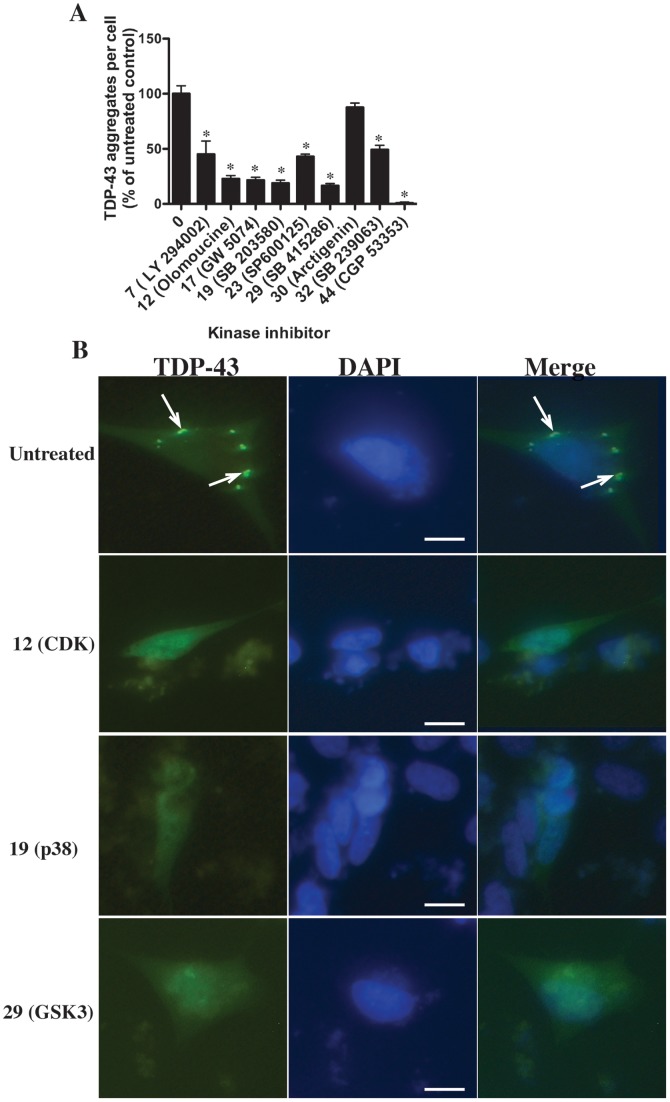
Effect of selected kinase inhibitors on aggregation of CTF-TDP-43-GFP (219–414). Non-retinoic acid-treated SH-SY5Y cells were transfected with CTF-TDP-43 (219-414)-GFP and incubated for 24 h. Selected kinase inhibitors were added for a further 24 h and the numbers of GFP-positive cytosolic inclusions determined and compared to untreated cells (no inhibitor) (**A**). The inhibitors examined were 10 µM LY294002 (#7, PI3K); olomoucine (#12, CDKs); GW 5074 (#17, Raf); SB 203580 (#19, p38); SP600125 (#23, JNK); (1 µM) SB 415286 (#29, GSK3); arctigenin (#30, MEK); (1 µM) SB 239063 (#32, p38); CGP 53353 (#44 PKC). *P<0.05 compared to untreated controls. **B:** Representative image of untreated and cells treated with olomoucine (#12, CDKs); SB 203580 (#19, p38); or SB 415286 (#29, GSK3). Green = TDP-43 (GFP), blue = DAPI, righthand column represents merged image of TDP-43 and DAPI. Arrows indicate cytosolic inclusions. Bar = 10 µm.

Finally, to obtain a further insight into the action of the selected kinase inhibitors on TDP-43 cytosolic accumulation, we examined the effect of the inhibitors on C-terminal TDP-43 219-414-GFP (CTF-TDP-43). C-terminal TDP-43 is the major accumulating form of TDP-43 in TDP-43 proteinopathies. We have previously shown that kinases may, at least partially, control C-terminal TDP-43 accumulation. Therefore, in this study we examined this further. As previously reported, non-differentiated SH-SY5Y cells transfected with CTF-TDP-43 revealed localized accumulation of TDP-43 within the cytosol without additional stress induction. Upon treatment with selected kinase inhibitors, we observed the inhibition of TDP-43 accumulation with LY294002 (#7, PI3K); olomoucine (#12, CDK); GW5074 (#17, Raf); SB 203580 (#19, p38); SP600125 (#23, JNK); SB 415286 (#29, GSK3); SB 239063 (#32, p38); and CGP 53353 (#44, PKC) (Figure 5). Interestingly, these findings demonstrated that there is significant overlap in the effect of selected CDK, GSK3 and Raf/MEK pathway inhibitors on cytosolic accumulation of endogenous TDP-43 and transfected CTF-TDP-43. As no stress was applied to the transfected cells, the results further demonstrate that either the expression and/or accumulation of CTF-TDP-43 alone is a cell stress inducer or the kinases identified in this study may be critical for controlling accumulation of TDP-43 but may not be specifically induced by stress alone. However, importantly, our study has identified several kinase pathways (PI3K, CDK, GSK3 and MAPK-associated pathways) that are important for control of TDP-43 and its interacting protein partners, hnRNP K and TIAR.

## Discussion

The accumulation of stress granule proteins such as hnRNPs, HuR and TIA-1 or TIAR are controlled by the action of protein kinases [13]–[18]. As TDP-43 is also a member of the hnRNP family, we investigated the role of protein kinases in TDP-43 accumulation in SH-SY5Y neuronal-like cultures subjected to stress using paraquat treatment as previously reported. Cells were co-treated cells with a range of kinase inhibitors from the Tocriscreen kinase inhibitor toolkit. Unsurprisingly, we identified a substantial number of kinase inhibitors that induced significant inhibition of TDP-43 and/or HuR accumulation. Although the large number of effects are beyond the scope of discussion here, some obvious patterns emerged. Inhibitors of the well-known PI3K and MAPK pathways including inhibitors of Raf, MEK/ERK, p38 and JNK consistently modulated (mostly inhibited) TDP-43 and/or HuR-positive stress granule formation from paraquat treatment. This is consistent with previous reports by ourselves and others that these kinase pathways are associated with control of hnRNPs [17], [19], [21]–[23]. Interestingly, only a few inhibitors induced increases in stress granule formation, suggesting that more kinases are associated with up-regulation of stress granule formation rather than its inhibition. Likewise, only inhibition of a few kinases (e.g., MLCK, PKB and Chk1) were found to result in loss of HuR-positive stress granules without loss of TDP-43 (and hnRNP K/TIAR)-positive stress granules. This perhaps indicates that HuR localization to stress granules is a global event while TDP-43 only occurs in a sub-set of stress granules. This has been supported by our previous studies showing that TDP-43 only occurs in a portion of HuR-positive stress granules [23] and studies reporting that HuR is a major stress granule component [42].

Due to the fact that most kinase inhibitors reveal only selectivity for particular kinase targets and are not specific for only one kinase, it can be problematic to identify a kinase associated with inhibition of TDP-43 accumulation based on a single inhibitor. However, for many of the kinase targets, there were multiple inhibitors tested and this can increase the likelihood that a specific target kinase is associated with TDP-43 accumulation. Using this approach we identified inhibitors of CDKs as a potentially important control point for TDP-43 cytoplasmic accumulation. We found that four inhibitors (olomoucine, arcyriaflavin A, ryuvidine and ZM 447439) blocked formation of TDP-43-positive stress granules without effect on HuR-positive stress granule formation. Two additional CDK inhibitors (purvalanol B and aminopurvalanol A) blocked formation of both TDP-43 and HuR-positive stress granule formation while two (purvalanol A and NSC 693868) had no effect at the concentrations tested. Notably, CDK inhibitors also reversed the accumulation of TDP-43-positive stress granules when added for the final 6 h of incubation. This strongly suggests that while JNK, other MAPKs and GSK3 are associated with formation of TDP-43-positive stress granules, CDKs may be essential in maintaining their formation once established.

The involvement of CDKs in modulating TDP-43 and additional stress granule protein accumulation is not well understood. Lian and Gallouzi reported that decreased levels of cyclin kinase inhibitor p21 in senescent cells was associated with increased stress granule formation [43]. More recently, it has been demonstrated that inhibition of cell division cycle 7 (CDC7) kinase also reduces TDP-43 phosphorylation and associated neurodegeneration in TDP-43 transgenic mice [44]. It is not clear whether this involves TDP-43 stress granule accumulation, however, notably, CDC7 and CDK2 are closely associated [45]. It has also been shown that loss of TDP-43 expression leads to altered CDK6 protein and transcript levels [46] although the specific control of hnRNPs by CDKs has not been well investigated. The CDK inhibitors used here have a potential to inhibit several members of the CDK family including CDK1, CDK2/4, and/or CDK5 [47], [48]. Additional studies are necessary to determine which of these CDKs control TDP-43 accumulation and their mechanism of action. However, we did show that CDKs were also associated with aggregation of CTF-TDP-43 transfected into cells suggesting that CDKs may control accumulation of C-terminal TDP-43 regardless of the origin of cell stress. This was further supported by the fact that CDK inhibition blocked TDP-43 accumulation in HeLa cells exposed to arsenite and SH-SY5Y cells exposed to paraquat. These findings, together with the report by Liachko et al., [44] on the CDC7 role in TDP-43 pathology clearly support a role for altered cell cycle protein homeostasis in abnormal TDP-43 metabolism.

GSK3 was also identified as a key kinase associated with formation of TDP-43-positive stress granules. Three inhibitors of GSK3 all reduced formation of TDP-43-positive stress granules and two of the three (SB 216763 and SB 415286) were specific for TDP-43 effects. GSK3 has an essential role in neurodegenerative disease and has long been a potential therapeutic target for treatment of Alzheimer’s disease, Parkinson’s disease, frontotemporal dementia and ALS [49]–[53]. The key role of GSK3 in these diseases is not yet fully understood but may be related to its ability to modulate cytoskeletal functions through phosphorylation of the microtubule protein tau [52]. Interestingly, microtubule function has been shown to be essential for TDP-43 accumulation [54] and inhibition of GSK3 blocked TDP-43 neurotoxicity in a *Drosophila* model. Microtubular integrity is also essential for stress granule dynamics [55]. Further studies are required to understand the role of GSK3 in TDP-43 accumulation, however, these studies support an important role for this kinase in TDP-43 processing.

One of the intriguing aspects of kinase control of TDP-43 is that the latter has no known consensus sites for phosphorylation by these kinases. Hasegawa et al. [56] reported that TDP-43 is potentially phosphorylated at multiple sites by casein kinase, including the phosphorylation site at 409/410 of C-terminal TDP-43. We have shown here that CK2 inhibition potentially modulates TDP-43 accumulation. It is possible that direct phosphorylation of TDP-43 may occur at non-consensus sites. It is also feasible that TDP-43 could be phosphorylated by unknown kinases or CK2 via regulation by JNK, GSK3 or CDKs. In fact Higashi et al., [57] recently reported that JNK-mediated phosphorylation of TDP-43 was responsible for accumulation in cells exposed to arsenite. It is not known if this was direct or via control of other kinases. Alternatively, the control of TDP-43 accumulation could be mediated via phosphorylation of known binding partner proteins such as hnRNPs and TIA-1/TIAR. hnRNP K has consensus sites for phosphorylation by JNK, ERK and CDKs, although not GSK3. It has also been demonstrated that JNK modulates hnRNP K accumulation through phosphorylation of the protein [58]. We reported previously that TDP-43 and hnRNP K were co-localized in SH-SY5Y cells exposed to stress and that was confirmed in this study. While specific interactions between TDP-43 and hnRNP K have not been robustly investigated beyond proteomic screening [59], considerable research has identified important interactions between TDP-43 and other members of the hnRNP family including hnRNP A/B [60], [61]. Recently, Mori et al [62] reported that a range of hnRNPs, including hnRNP K and hnRNP A3 interacted with the hexanucleotide repeat region of C9orf72 mRNA, which has been linked to large numbers of ALS and FTLD cases. Moreover, hnRNP A3 was found to be aggregated in cytosolic inclusions in the brains of patients with C9orf72 expanded repeats. Although these inclusions were TDP-43 negative, this study provides strong support for the role of alternative hnRNP molecules in ALS and FTLD. As hnRNPs are known to be controlled by kinases, as supported by our data here, specific kinases could modulate TDP-43 accumulation via interaction with other hnRNPs. Studies have also shown that TIA-1 interacts with TDP-43 [7] and this protein has phosphorylation consensus sites for JNK but not CDKs suggesting that TIA-1 or TIAR could also control TDP-43 accumulation. However, further support for TDP-43/hnRNP or TIA-1 interactions will require extensive studies involving knockdown of the proteins in question, together with investigation of the putative phosphorylation sites and how these modulate interaction with, and movement of, TDP-43.

In summary, we have used an *in vitro* model of TDP-43-positive stress granule formation to screen for the effect of kinase inhibitors on TDP-43 accumulation. We have found that while a number of kinase inhibitors, particularly of the MAPK pathways, modulated both TDP-43 and the global stress granule marker, HuR, multiple inhibitors also more specifically targeted TDP-43 accumulation, including inhibitors of CDKs and GSK3. Close correlation was observed between effects of these inhibitors on TDP-43, hnRNP K and TIAR while differential effects were often observed on HuR accumulation. Further examination of selected inhibitors also revealed a high level of conservation of TDP-43-controlling kinase pathways in different cell types and stresses. Moreover, CDK inhibitors were found to reverse pre-formed TDP-43-positive stress granules rather than just preventing their initial formation. Inhibition of CTF-TDP-43 accumulation in transfected cells with CDK and GSK3 inhibitors suggests a role for modulation of C-terminal TDP-43 trafficking by the identified kinase groups. Further studies are required to confirm the specific kinases involved and whether their action is through phosphorylation of the TDP-43 binding partners, hnRNP K and/or TIAR. This knowledge provides a valuable insight into the mechanisms controlling abnormal cytoplasmic TDP-43 accumulation that is a hallmark of TDP-43 proteinopathies such as ALS and FTLD and may herald new opportunities for kinase modulation-based therapeutic intervention in these diseases.

## Supporting Information

Figure S1 Effect of selected kinase inhibitors on TDP-43 and HuR-positive stress granule formation. SH-SY5Y cells were treated with 1 mM paraquat (PQ) overnight in the presence or absence of 10 µM LY294002 (#7, PI3K); 10 µM U0126 (#8, MEK); 10 µM olomoucine (#12, CDKs); 10 µM SB 415286 (#29, GSK3); or 10 µM SB 239063 (#32, p38). Green = TDP-43, red = HuR, blue = DAPI. Righthand column shows merged images of TDP-43 and HuR. Arrows indicate stress granules common to both TDP-43 and HuR images. Closed arrowheads indicate HuR-specific stress granules. Open arrowheads indicate cytosolic diffuse TDP-43. Bar = 10 µm.
(TIF)

Figure S2 Effect of additional selected kinase inhibitors on TDP-43 and HuR-positive stress granule formation. SH-SY5Y cells were treated with 1 mM paraquat (PQ) overnight in the presence or absence of 10 µM PD98059 (#9, MEK); 10 µM GW5074 (#17, Raf); 10 µM SB 203580 (#19, 038); or 10 µM Arctigenin (#30, MEK). Green = TDP-43, red = HuR, blue = DAPI. Righthand column shows merged images of TDP-43 and HuR. Arrows indicate stress granules common to both TDP-43 and HuR images. Bar = 10 µm.
(TIF)

Figure S3 Effect of additional selected kinase inhibitors on TDP-43 and HuR-positive stress granule formation. SH-SY5Y cells were treated with 1 mM paraquat (PQ) overnight in the presence or absence of 1 µM aminopuvalonol A (#35, CDKs); 10 µM HA 1100 (#42, ROCK); 10 µM CGP 533353 (#44, PKC); 10 µM arcyriaflavin A (#45, CDK); or 10 µM ZM 447439 (#46, Aurora). Green = TDP-43, red = HuR, blue = DAPI. Righthand column shows merged images of TDP-43 and HuR. Arrows indicate stress granules common to both TDP-43 and HuR images. Closed arrowheads indicate HuR-specific stress granules. Bar = 10 µm.
(TIF)

Figure S4 Effect of selected kinase inhibitors on TDP-43 expression. SH-SY5Y cells were treated with paraquat (PQ) overnight in the presence or absence of 10 µM LY294002 (#7, PI3K); olomoucine (#12, CDKs); ZM 449829 (#15, JAK3); GW 5074 (#17, Raf); SB 203580 (#19, p38); SB 415286 (#29, GSK3); arctigenin (#30, MEK); SB 239063 (#32, p38); (1 µM) aminopurvalanol A (#35, CDKs); TBB (#40, CK2); HA 1100 (#42, ROCK); BIBX 1382 (#43, EGFR); CGP 53353 (#44, PKC); arcyriaflavin A (#45, CDKs). Western blot analysis of TDP-43 expression was determined compared to GAPDH control. Representative image from three experiments. Dotted lines indicate removal of unrelated lanes.
(TIF)

Figure S5 Effect of selected kinase inhibitors on TDP-43 and hnRNP K-positive stress granule formation. SH-SY5Y cells were treated with 1 mM paraquat (PQ) overnight in the presence or absence of 10 µM LY294002 (#7, PI3K); 10 µM U0126 (#8, MEK);10 µM olomoucine (#12, CDKs); 10 µM SB 415286 (#29, GSK3); or 10 µM SB 299063 (#32, p38). Green = TDP-43, red = hnRNP K, blue = DAPI. Righthand column shows merged images of TDP-43 and hnRNP K. Arrows indicate stress granules common to both TDP-43 and hnRNP K images. Bar = 10 µm.
(TIF)

Figure S6 Effect of selected kinase inhibitors on TDP-43 and TIAR-positive stress granule formation. SH-SY5Y cells were treated with 1 mM paraquat (PQ) overnight in the presence or absence of 10 µM LY294002 (#7, PI3K); 10 µM olomoucine (#12, CDKs); or 10 µM SB 415286 (#29, GSK3). Green = TDP-43, red = TIAR, blue = DAPI. Righthand column shows merged images of TDP-43 and TIAR. Arrows indicate stress granules common to both TDP-43 and TIAR images. Bar = 10 µm.
(TIF)

Figure S7 Effect of CDK inhibitors on hnRNP A1 accumulation in paraquat-treated cells. SH-SY5Y cells were treated with 1 mM paraquat (PQ) overnight with or without 10 µM of the CDK inhibitors olomoucine (#12) or arcyriaflavin A (#45). Green = hnRNP A1, blue = DAPI. Righthand column shows merged images of hnRNP A1 and DAPI. Bar = 10 µm.
(TIF)

Figure S8 Stress granule inhibition by olomoucine (#12) CDK inhibitor in retinoic acid-treated and non-treated SH-SY5Y cells. SH-SY5Y cells were treated with retinoic acid or left un-treated as described in Methods. Cells were then treated overnight with 1 mM paraquat (PQ) in the presence or absence of 10 µM olomoucine. Green = TDP-43, blue = DAPI. Arrows indicate TDP-43-positive stress granules. Bar = 10 µm.
(TIF)

Figure S9 Stress granule inhibition by olomoucine (#12) CDK inhibitor in HeLa cells. HeLa cells were treated overnight with 50 µM sodium arsenite overnight in the presence or absence of 10 µM olomoucine. Green = TDP-43, red = HuR, blue = DAPI. Righthand column shows merged images of TDP-43 and HuR. Arrows indicate TDP-43-positive stress granules. Arrowhead indicates HuR-specific stress granule. Bar = 10 µm.
(TIF)

Figure S10 Effect of selected kinase inhibitors on reversal of pre-formed TDP-43 and HuR-positive stress granules. SH-SY5Y cells were treated with 1 mM paraquat (PQ) overnight and exposed to selected inhibitors for the final 6 h of incubation (10 µM olomoucine (#12, CDKs); or 10 µM arcyriaflavin A (#45, CDKs). Green = TDP-43, red = HuR, blue = DAPI. Righthand column shows merged images of TDP-43 and HuR. Arrows indicate stress granules. Bar = 10 µm.
(TIF)

Table S1 List of kinase abbreviations and names.
(DOCX)

Table S2 Effect of kinase inhibitors on TDP-43 and HuR-positive stress granule formation induced by paraquat treatment in SH-SY5Y cells.
(DOCX)

Table S3 List of kinase classes associated with formation of TDP-43 and/or HuR-positive stress granules induced by paraquat treatment in SH-SY5Y cells.
(DOCX)

Table S4 Dose-response effect of representative kinase inhibitors on TDP-43-positive stress granules and inhibition of target kinase phosphorylation.
(DOCX)

Table S5 Effect of kinase inhibitors on TDP-43, hnRNP K, TIAR and HuR-positive stress granule formation induced by paraquat treatment in SH-SY5Y cells.
(DOCX)

Table S6 Comparison of kinases associated with formation of TDP-43, hnRNP K, TIAR and/or HuR-positive stress granules induced by paraquat treatment in SH-SY5Y cells.
(DOCX)

Table S7 Effect of kinase inhibitors on formation of TDP-43-positive stress granules induced by paraquat treatment in retinoic-acid treated compared to non-treated SH-SY5Y cells.
(DOCX)

Table S8 Effect of kinase inhibitors on formation of TDP-43 and HuR-positive stress granules induced by sodium arsenite treatment in HeLa epithelial cells.
(DOCX)

Table S9 Ability of kinase inhibitors to reverse TDP-43 and/or HuR-positive stress granule formation induced by paraquat treatment when for the final 6 hr of 24 hr paraquat treatment (SH-SY5Y cells).
(DOCX)
