# Supplementary figures and images for: Kinase Inhibitor Screening Identifies Cyclin-Dependent Kinases and Glycogen Synthase Kinase 3 as Potential Modulators of TDP-43 Cytosolic Accumulation during Cell Stress
Source: PLoS One. 2013 Jun 26;8(6):e67433. doi: 10.1371/journal.pone.0067433 (PMC3694067; doi:10.1371/journal.pone.0067433)

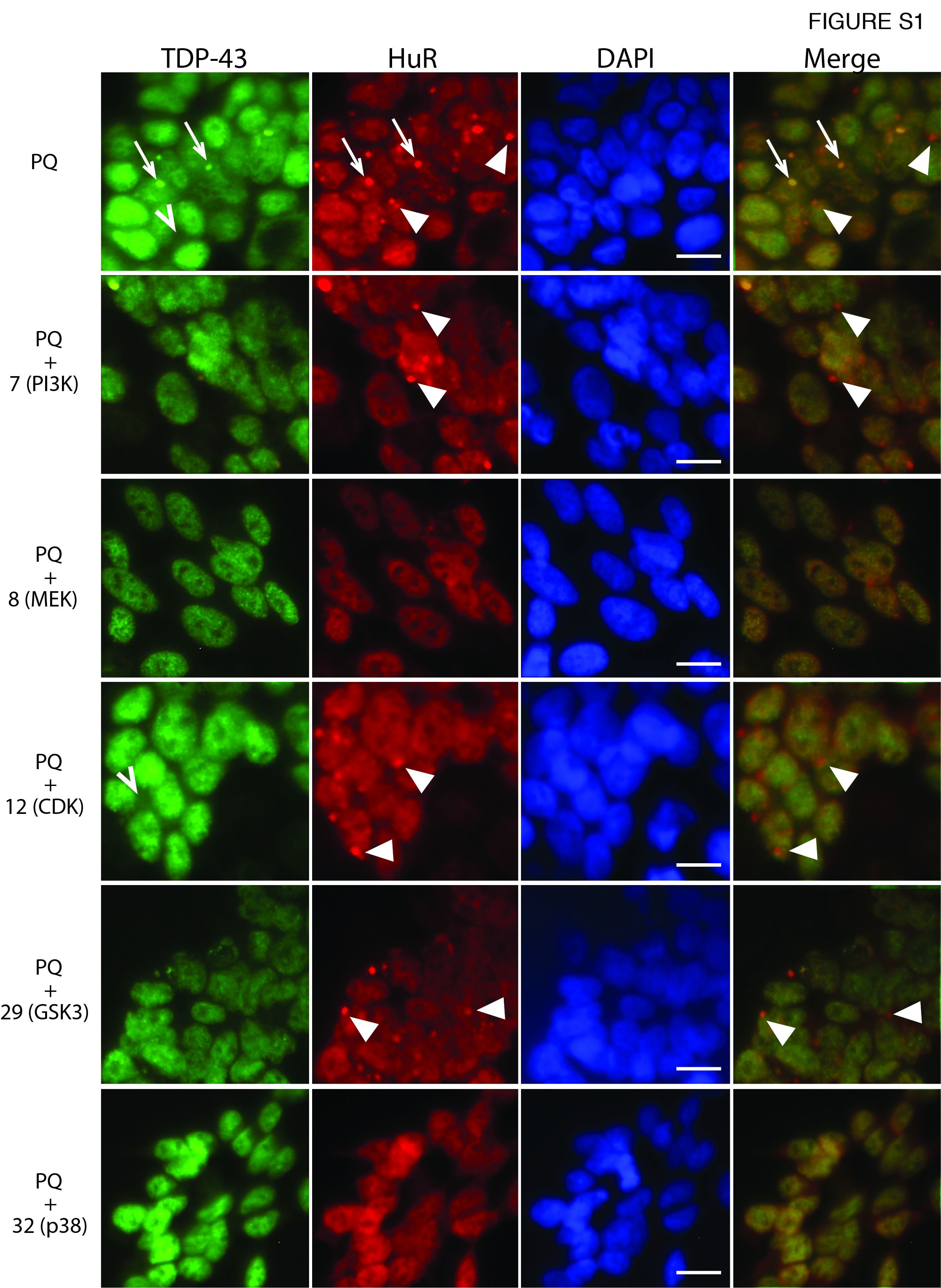

Supplement: Figure S1 — Effect of selected kinase inhibitors on TDP-43 and HuR-positive stress granule formation. SH-SY5Y cells were treated with 1 mM paraquat (PQ) overnight in the presence or absence of 10 µM LY294002 (#7, PI3K); 10 µM U0126 (#8, MEK); 10 µM olomoucine (#12, CDKs); 10 µM SB 415286 (#29, GSK3); or 10 µM SB 239063 (#32, p38). Green = TDP-43, red = HuR, blue = DAPI. Righthand column shows merged images of TDP-43 and HuR. Arrows indicate stress granules common to both TDP-43 and HuR images. Closed arrowheads indicate HuR-specific stress granules. Open arrowheads indicate cytosolic diffuse TDP-43. Bar = 10 µm. (TIF) [file pone.0067433.s001.tif]

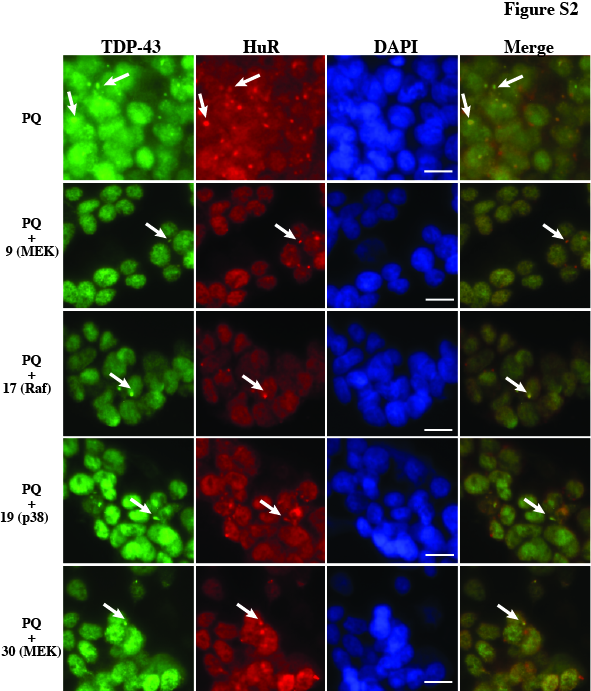

Supplement: Figure S2 — Effect of additional selected kinase inhibitors on TDP-43 and HuR-positive stress granule formation. SH-SY5Y cells were treated with 1 mM paraquat (PQ) overnight in the presence or absence of 10 µM PD98059 (#9, MEK); 10 µM GW5074 (#17, Raf); 10 µM SB 203580 (#19, 038); or 10 µM Arctigenin (#30, MEK). Green = TDP-43, red = HuR, blue = DAPI. Righthand column shows merged images of TDP-43 and HuR. Arrows indicate stress granules common to both TDP-43 and HuR images. Bar = 10 µm. (TIF) [file pone.0067433.s002.tif]

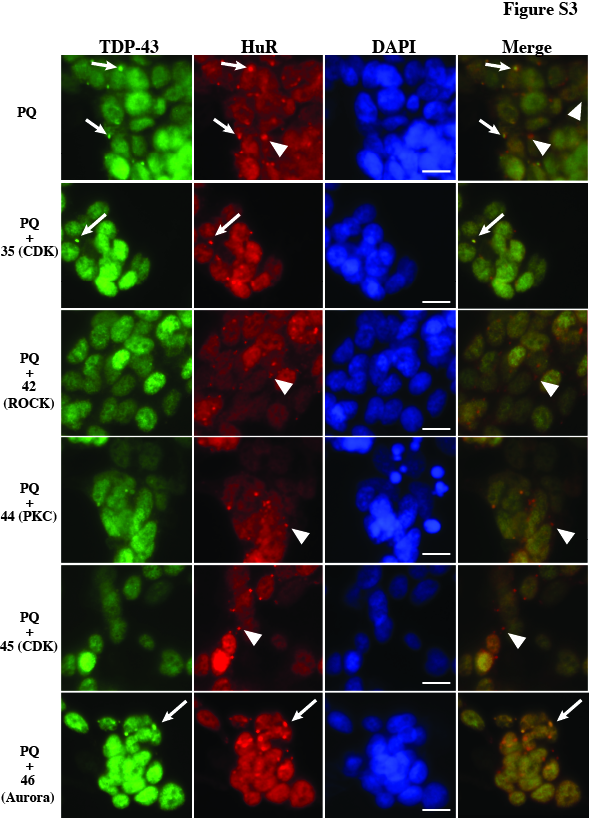

Supplement: Figure S3 — Effect of additional selected kinase inhibitors on TDP-43 and HuR-positive stress granule formation. SH-SY5Y cells were treated with 1 mM paraquat (PQ) overnight in the presence or absence of 1 µM aminopuvalonol A (#35, CDKs); 10 µM HA 1100 (#42, ROCK); 10 µM CGP 533353 (#44, PKC); 10 µM arcyriaflavin A (#45, CDK); or 10 µM ZM 447439 (#46, Aurora). Green = TDP-43, red = HuR, blue = DAPI. Righthand column shows merged images of TDP-43 and HuR. Arrows indicate stress granules common to both TDP-43 and HuR images. Closed arrowheads indicate HuR-specific stress granules. Bar = 10 µm. (TIF) [file pone.0067433.s003.tif]

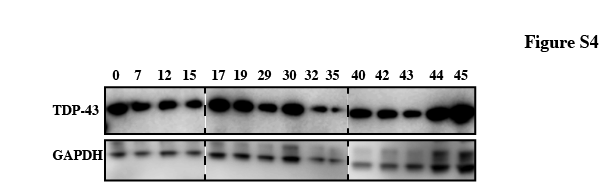

Supplement: Figure S4 — Effect of selected kinase inhibitors on TDP-43 expression. SH-SY5Y cells were treated with paraquat (PQ) overnight in the presence or absence of 10 µM LY294002 (#7, PI3K); olomoucine (#12, CDKs); ZM 449829 (#15, JAK3); GW 5074 (#17, Raf); SB 203580 (#19, p38); SB 415286 (#29, GSK3); arctigenin (#30, MEK); SB 239063 (#32, p38); (1 µM) aminopurvalanol A (#35, CDKs); TBB (#40, CK2); HA 1100 (#42, ROCK); BIBX 1382 (#43, EGFR); CGP 53353 (#44, PKC); arcyriaflavin A (#45, CDKs). Western blot analysis of TDP-43 expression was determined compared to GAPDH control. Representative image from three experiments. Dotted lines indicate removal of unrelated lanes. (TIF) [file pone.0067433.s004.tif]

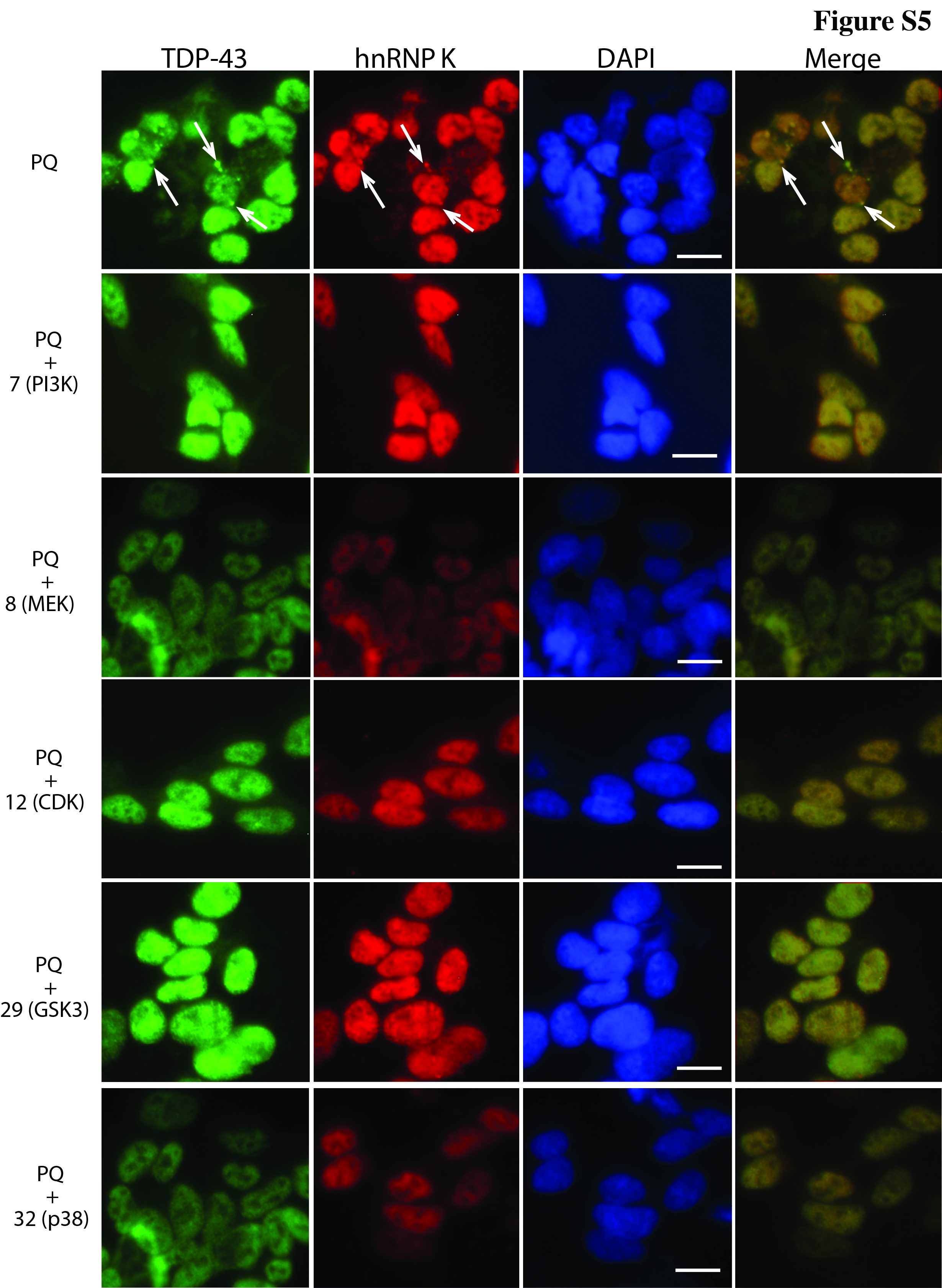

Supplement: Figure S5 — Effect of selected kinase inhibitors on TDP-43 and hnRNP K-positive stress granule formation. SH-SY5Y cells were treated with 1 mM paraquat (PQ) overnight in the presence or absence of 10 µM LY294002 (#7, PI3K); 10 µM U0126 (#8, MEK);10 µM olomoucine (#12, CDKs); 10 µM SB 415286 (#29, GSK3); or 10 µM SB 299063 (#32, p38). Green = TDP-43, red = hnRNP K, blue = DAPI. Righthand column shows merged images of TDP-43 and hnRNP K. Arrows indicate stress granules common to both TDP-43 and hnRNP K images. Bar = 10 µm. (TIF) [file pone.0067433.s005.tif]

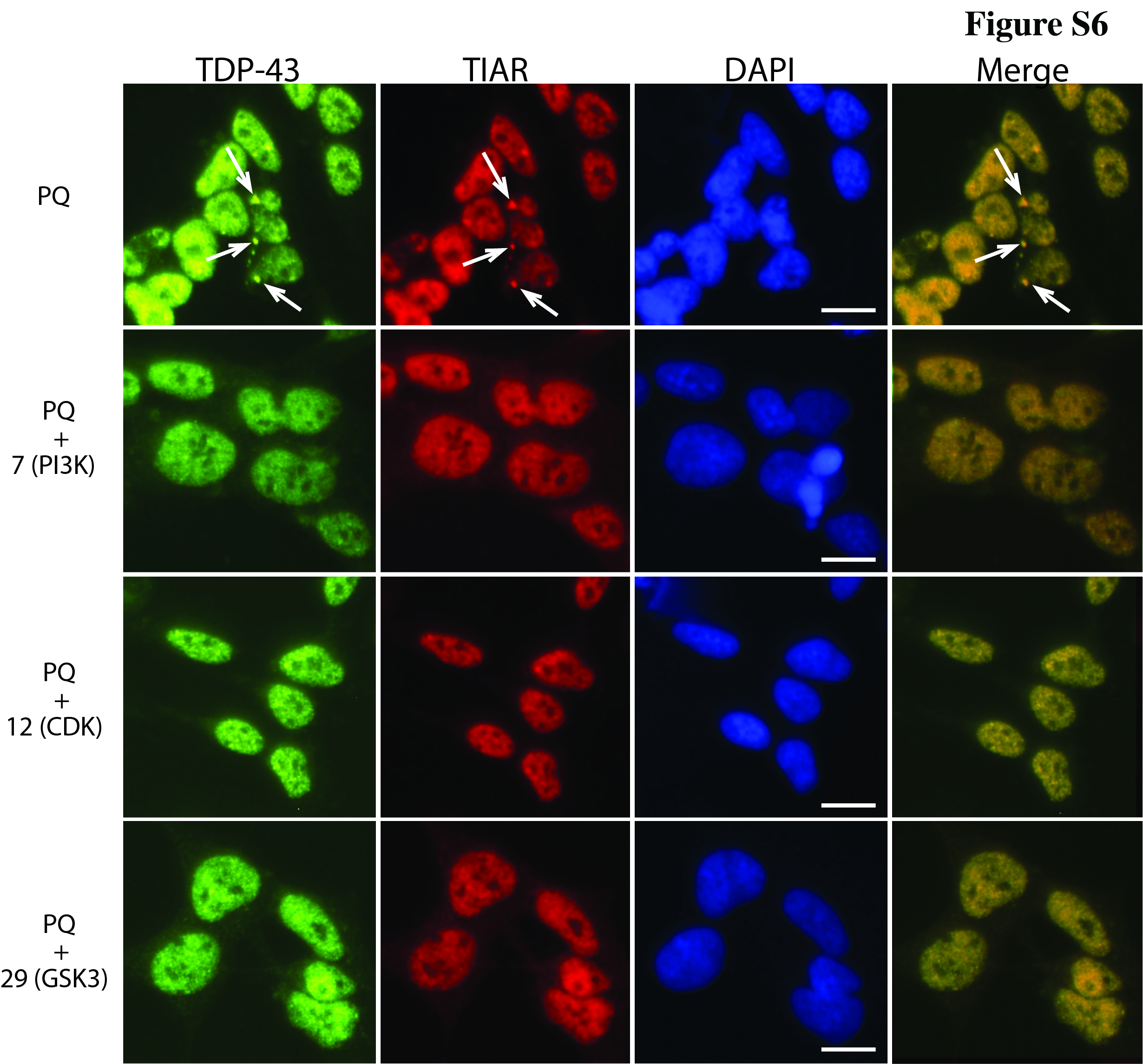

Supplement: Figure S6 — Effect of selected kinase inhibitors on TDP-43 and TIAR-positive stress granule formation. SH-SY5Y cells were treated with 1 mM paraquat (PQ) overnight in the presence or absence of 10 µM LY294002 (#7, PI3K); 10 µM olomoucine (#12, CDKs); or 10 µM SB 415286 (#29, GSK3). Green = TDP-43, red = TIAR, blue = DAPI. Righthand column shows merged images of TDP-43 and TIAR. Arrows indicate stress granules common to both TDP-43 and TIAR images. Bar = 10 µm. (TIF) [file pone.0067433.s006.tif]

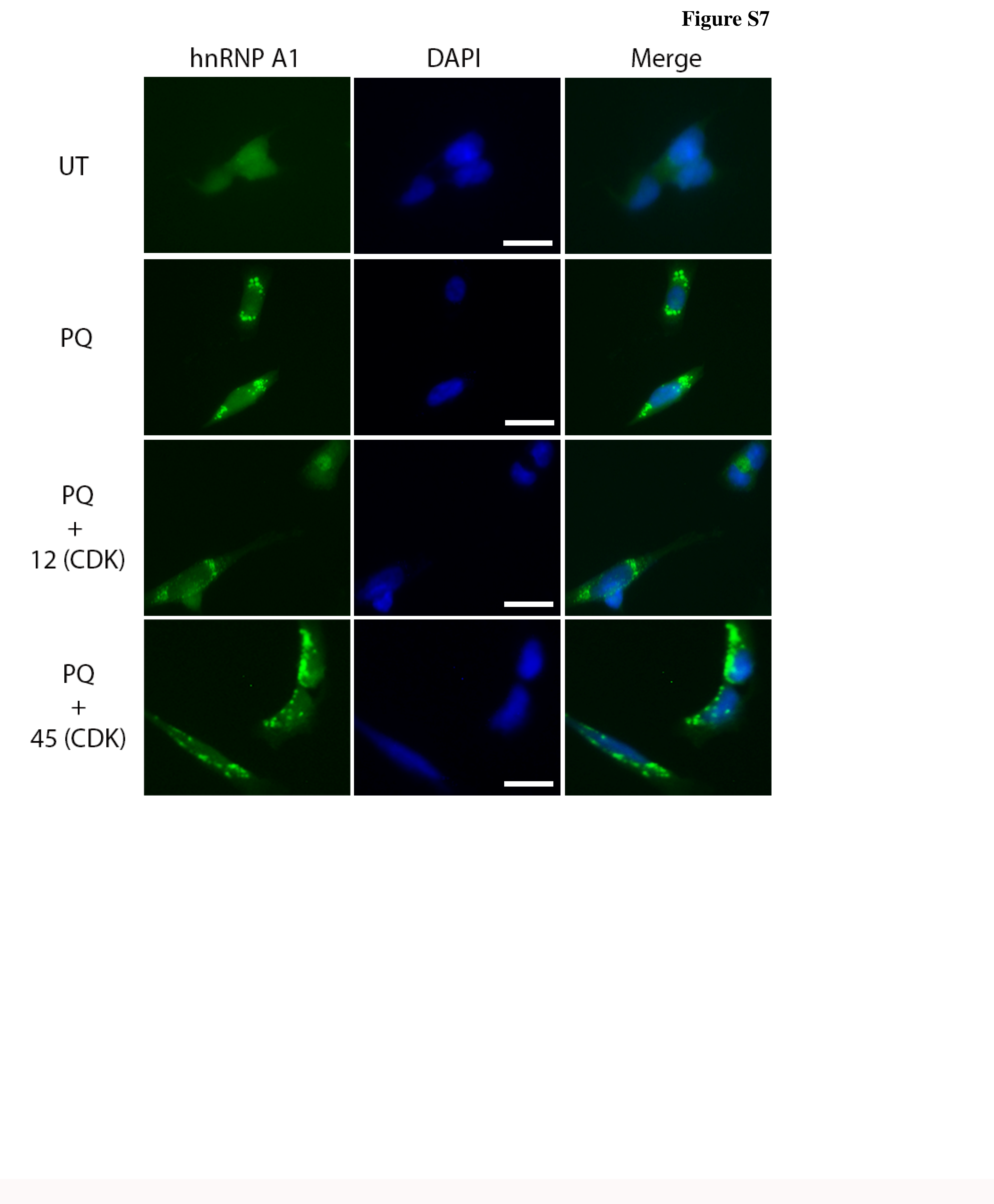

Supplement: Figure S7 — Effect of CDK inhibitors on hnRNP A1 accumulation in paraquat-treated cells. SH-SY5Y cells were treated with 1 mM paraquat (PQ) overnight with or without 10 µM of the CDK inhibitors olomoucine (#12) or arcyriaflavin A (#45). Green = hnRNP A1, blue = DAPI. Righthand column shows merged images of hnRNP A1 and DAPI. Bar = 10 µm. (TIF) [file pone.0067433.s007.tif]

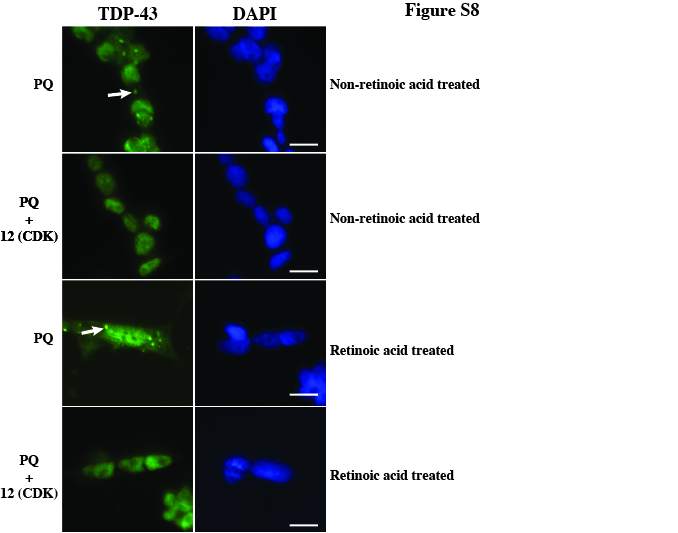

Supplement: Figure S8 — Stress granule inhibition by olomoucine (#12) CDK inhibitor in retinoic acid-treated and non-treated SH-SY5Y cells. SH-SY5Y cells were treated with retinoic acid or left un-treated as described in Methods. Cells were then treated overnight with 1 mM paraquat (PQ) in the presence or absence of 10 µM olomoucine. Green = TDP-43, blue = DAPI. Arrows indicate TDP-43-positive stress granules. Bar = 10 µm. (TIF) [file pone.0067433.s008.tif]

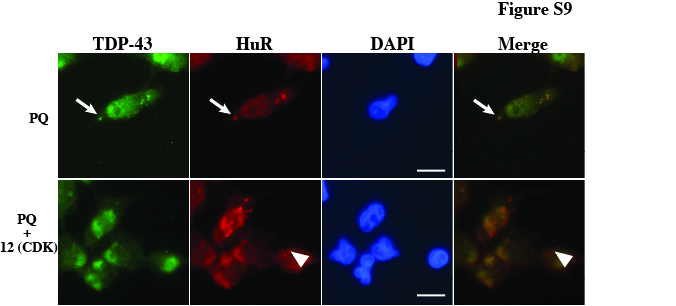

Supplement: Figure S9 — Stress granule inhibition by olomoucine (#12) CDK inhibitor in HeLa cells. HeLa cells were treated overnight with 50 µM sodium arsenite overnight in the presence or absence of 10 µM olomoucine. Green = TDP-43, red = HuR, blue = DAPI. Righthand column shows merged images of TDP-43 and HuR. Arrows indicate TDP-43-positive stress granules. Arrowhead indicates HuR-specific stress granule. Bar = 10 µm. (TIF) [file pone.0067433.s009.tif]

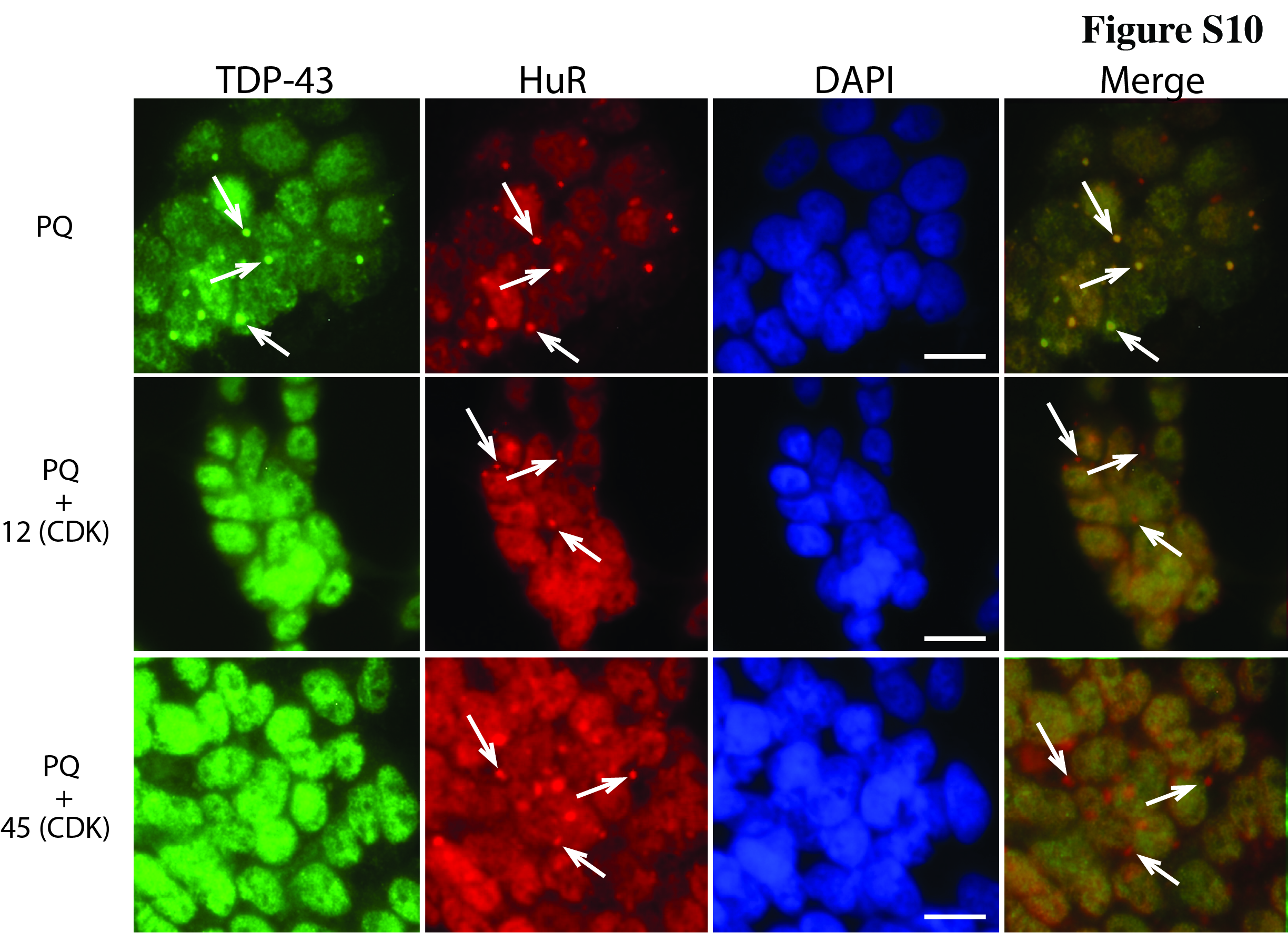

Supplement: Figure S10 — Effect of selected kinase inhibitors on reversal of pre-formed TDP-43 and HuR-positive stress granules. SH-SY5Y cells were treated with 1 mM paraquat (PQ) overnight and exposed to selected inhibitors for the final 6 h of incubation (10 µM olomoucine (#12, CDKs); or 10 µM arcyriaflavin A (#45, CDKs). Green = TDP-43, red = HuR, blue = DAPI. Righthand column shows merged images of TDP-43 and HuR. Arrows indicate stress granules. Bar = 10 µm. (TIF) [file pone.0067433.s010.tif]
