# Supplementary material for: Kinase Inhibitor Screening Identifies Cyclin-Dependent Kinases and Glycogen Synthase Kinase 3 as Potential Modulators of TDP-43 Cytosolic Accumulation during Cell Stress
Source: PLoS One. 2013 Jun 26;8(6):e67433. doi: 10.1371/journal.pone.0067433 (PMC3694067; doi:10.1371/journal.pone.0067433)
Supplement: Table S1 — List of kinase abbreviations and names. (DOCX) [file pone.0067433.s011.docx]

**Table S1:** List of kinase abbreviations and names.

| **Kinase inhibitor number** | **Kinase inhibitor name** | **Target kinase abbreviation** | **Target kinase name** |
| --- | --- | --- | --- |
| 1 | AG 490 | EGFR | Epidermal growth factor inhibitor |
| 2 | ML 9 hydrochloride | MLCK | Mysosin light chain kinase |
| 4 | Fasudil hydrochloride | ROCK | Rho kinase |
| 5 | GF 109203X | PKC | Protein kinase C |
| 7 | LY 294002 hydrochloride | PI3K | Phosphoinositol-3-kinase |
| 8 | U0126 | MEK | Mitogen-activated protein kinase kinase |
| 11 | SB 202190 | p38 MAPK | p38 mitogen activated protein kinase |
| 12 | Olomoucine | CDK | Cyclin-dependent kinase |
| 13 | LFM-A13 | BTK | Bruton’s tyrosine kinase |
| 15 | ZM 449829 | JAK3 | Janus kinase 3 |
| 22 | SU 4312 | VEGFR | Vascular endothelial growth factor receptor |
| 23 | SP 600125 | JNK | c-Jun N-terminal kinase |
| 27 | SB 431542 | TGFbR1 | Transforming growth factor, beta receptor I |
| 28 | SB 216763 | GSK-3 | Glycogen synthase kinase 3 |
| 36 | API-2 | PKB | Protein kinase B |
| 40 | TBB | CK2 | Casein kinase 2 |
| 41 | 1,2,3,4,5,6-Hexabromocyclohexane | JAK2 | Janus kinase 2 |
| 50 | IKK 16 | IKK | I Kappa B kinase |
| 55 | TCS 359 | FLT3 | Fms-like tyrosine kinase 3 |
| 59 | CGK 733 | ATR/ATM | Ataxia telangiectasia and Rad3-related protein/ataxia telangiectasia, mutated |
| 64 | PQ 401 | IGF-1R | Insulin-like growth factor 1 receptor |
| 66 | NU 7026 | DNA-PK | DNA-protein kinase |
| 67 | D 4476 | CK1 | Casein kinase inhibitor 1 |
| 69 | H 89 dihydrochloride | PKA | Protein kinase A |
| 71 | GW 843682X | PLK | Polo-like kinase |
| 75 | Dorsomorphin dihydrochloride | AMPK | AMP-activated protein kinase |
