# Supplementary material for: Kinase Inhibitor Screening Identifies Cyclin-Dependent Kinases and Glycogen Synthase Kinase 3 as Potential Modulators of TDP-43 Cytosolic Accumulation during Cell Stress
Source: PLoS One. 2013 Jun 26;8(6):e67433. doi: 10.1371/journal.pone.0067433 (PMC3694067; doi:10.1371/journal.pone.0067433)
Supplement: Table S2 — Effect of kinase inhibitors on TDP-43 and HuR-positive stress granule formation induced by paraquat treatment in SH-SY5Y cells. (DOCX) [file pone.0067433.s012.docx]

**Table S2:** Effect of kinase inhibitors on TDP-43 and HuR-positive stress granule formation induced by paraquat treatment in SH-SY5Y cells.

| **Kinase inhibitor number** | **Kinase inhibitor name** | **Target kinase** | **TDP-43 stress granule-positive cells**  **(% of paraquat treated cells)** | **Inhibition of TDP-43 stress granules (P<0.05 compared to control, Yes/No)** | **HuR stress granule-positive cells**  **(% of paraquat treated cells)** | **Inhibition of HuR stress granules (P<0.05 compared to control, Yes/No)** |
| --- | --- | --- | --- | --- | --- | --- |
| **0** | **Paraquat only** | **-** | **100 ± 4.4** | **NA** | **100 ± 2.6** | **NA** |
| 1 | AG 490 | EGFR | 147.4 ± 3.7 | No | 103.1 ± 7.4 | No |
| 2 | ML 9 hydrochloride | MLCK | 97.3 ± 3.3 | No | 77.4 ± 3 | No |
| 3 | AG 213 | EGFR | 104 ± 2.1 | No | 101 ± 1.4 | No |
| 4 | Fasudil hydrochloride | ROCK | 100 ± 2.3 | No | 107.9 ± 8.3 | No |
| 5 | GF 109203X | PKC | 73.8 ± 6.3 | No | 78.4 ± 3.2 | No |
| 6 | Genistein | EGFR | 64.1 ± 3.9 | Yes | 74 ± 11.2 | No |
| 7 | LY 294002 hydrochloride | PI3K | 36.8 ± 5 | Yes | 97.7 ± 3.4 | No |
| 8 | U0126 | MEK | 12.3 ± 4.7 | Yes | 27.2 ± 8.5 | Yes |
| 9 | PD 98059 | MEK | 18 ± 2.9 | Yes | 14.6 = 6.3 | Yes |
| 10 | Y-27632 dihydrochloride | ROCK | 94.8 ± 7.3 | No | 96.6 ± 9.3 | No |
| 11 | SB 202190 | p38 MAPK | 152.5 ± 5.6 | No | 105 ± 3.3 | No |
| 12 | Olomoucine | CDK | 6.6 ± 2.8 | Yes | 98.2 ± 2.4 | No |
| 13 | LFM-A13 | BTK | 137.5 ± 8.3 | No | 151 ± 13.9 | No |
| 14 | ZM 336372 | Raf | 43.1 ± 1.9 | Yes | 47.7 ± 3.6 | Yes |
| 15 | ZM 449829 | JAK3 | 72.2 ± 7.1 | No | 81.2 ± 6.9 | No |
| 16 | ZM 39923 hydrochloride | JAK3 | 93 ± 6.8 | No | 99.3 ± 4.1 | No |
| 17 | GW 5074 | Raf | 19.2 ± 2.6 | Yes | 5.4 ± 0.6 | Yes |
| 18 | PP 1 | Src | 102.6 ± 7.9 | No | 107.7 ± 5 | No |
| 19 | SB 203580 hydrochloride | p38 MAPK | 62.9 ± 13.7 | Yes | 55.1 ± 11.6 | Yes |
| 20^#^ | (-)-Terreic acid | BTK | 101.4 ± 6 | No | 103.1 ± 5.6 | No |
| 21^#^ | PP 2 | Src | Toxic | NA | Toxic | NA |
| 22 | SU 4312 | VEGFR | 100 ± 4 | No | 109.5 ± 13.2 | No |
| 23 | SP 600125 | JNK | 2.4 ± 1.7 | Yes | 97.2 ± 6.6 | No |
| 24^#^ | Purvalanol A | CDK | 96.1 ± 2 | No | 95.4 ± 1.8 | No |
| 25 | Purvalanol B | CDK | 37.7 ± 5.8 | Yes | 63.2 ± 7.8 | Yes |
| 26^#^ | Rottlerin | PKC | 102.4 ± 4.6 | No | 101 ± 8.2 | No |
| 27 | SB 431542 | TGFbR1 | 110 ± 5.1 | No | 105.7 ± 4.4 | No |
| 28 | SB 216763 | GSK-3 | 29.3 ± 2 | Yes | 103.2 ± 3.8 | No |
| 29^#^ | SB 415286 | GSK-3 | 9 ± 4.4 | Yes | 97 ± 2 | No |
| 30 | Arctigenin | MEK | 42.7 ± 4.3 | Yes | 43.3 ± 1.7 | Yes |
| 31 | NSC 693868 | CDK | 101 ± 3.9 | No | 97.8 ± 2 | No |
| 32^#^ | SB 239063 | p38 MAPK | 13.9 ± 3.7 | Yes | 18.2 ± 6.3 | Yes |
| 33 | SL 327 | MEK | 29.6 ± 8.8 | Yes | 38.3 ± 9.3 | Yes |
| 34 | Ro 31-8220 mesylate | Broad Spectrum Inhibitor | 145.2 ± 9.1 | No | 112 ± 14.7 | No |
| 35^#^ | Aminopurvalanol A | CDK | 2.8 ± 1.7 | Yes | 34 ± 1.8 | Yes |
| 36^#^ | API-2 | PKB | 105.1 ± 2.1 | No | 107.8 ± 2.6 | No |
| 37 | GW 441756 | TrkA | 107 ± 4.9 | No | 139.2 ± 1.6 | No |
| 38 | GW 583340 dihydrochloride | EGFR | 103.7 ± 5.7 | No | 95.1 ± 6.7 | No |
| 39 | Ro 08-2750 | TrkA | 249.1 ± 18.3 | No | 278.2 ± 23.4 | No |
| 40 | TBB | CK2 | 8.8 ± 5.3 | Yes | 128.1 ± 8.5 | No |
| 41 | 1,2,3,4,5,6-Hexabromocyclohexane | JAK2 | 106.7 ± 8.8 | No | 102 ± 7.7 | No |
| 42 | HA 1100 hydrochloride | ROCK | 15.3 ± 7.3 | Yes | 118.2 ± 12.8 | No |
| 43 | BIBX 1382 dihydrochloride | EGFR | 11.1 ± 9.4 | Yes | 93.7 ± 9 | No |
| 44 | CGP 53353 | PKC | 33 ± 3 | Yes | 100 ± 2 | No |
| 45 | Arcyriaflavin A | CDK | 2.7 ± 1.5 | Yes | 126.3 ± 14.9 | No |
| 46 | ZM 447439 | Aurora | 55.5 ± 11.2 | Yes | 107.2 ± 12 | No |
| 47^#^ | ER 27319 maleate | Syk | Toxic | NA | Toxic | NA |
| 48 | ZM 323881 hydrochloride | VEGFR | 97.9 ± 4.4 | No | 99 ± 9.1 | No |
| 49 | ZM 306416 hydrochloride | VEGFR | 92.1 ± 2.2 | No | 93.2 ± 2.9 | No |
| 50^#^ | IKK 16 | IKK | Toxic | NA | Toxic | NA |
| 51 | Ki 8751 | VEGFR | 105.2 ± 4 | No | 100.4 ± 6.7 | No |
| 52 | 10-DEBC hydrochloride | PKB | 100.1 ± 8.7 | No | 64.3 ± 8.1 | Yes |
| 53 | TPCA-1 | IKK | 100 ± 8.5 | No | 108.8 ± 4.7 | No |
| 54 | SB 218078 | Chk1 | 102.8 ± 7.3 | No | 71.7 ± 7.3 | No |
| 55 | TCS 359 | FLT3 | 106.6 ± 3.2 | No | 103 ± 4.5 | No |
| 56^#^ | PD 198306 | MEK | 144 ± 9.9 | No | 98.4 ± 8.3 | No |
| 57^#^ | Ryuvidine | CDK | 22.3 ± 6.5 | Yes | 101.6 ± 4.2 | No |
| 58^#^ | IMD 0354 | IKK | 104 ± 3.7 | No | 103 ± 9.9 | No |
| 59^#^ | CGK 733 | ATR/ATM | 99.6 ± 9 | No | 103.6 ± 7.3 | No |
| 60^#^ | PHA 665752 | cMET | * | NA | * | NA |
| 61^#^ | PD 407824 | Chk1 | 99.3 ± 2.6 | No | 97.4 ± 9.8 | No |
| 62 | LY 364947 | TGFbR1 | 94 ± 1.3 | No | 94.4 ± 5.6 | No |
| 63 | CGP 57380 | Mnk1 | 136.6 ± 12.4 | No | 99.1 ± 5.2 | No |
| 64 | PQ 401 | IGF-1R | 154.7 ± 12.9 | No | 90.5 ± 4.9 | No |
| 65 | PI 828 | PI3K | 73.2 ± 1.1 | No | 103.2 ± 9 | No |
| 66 | NU 7026 | DNA-PK | 96.7 ± 6.3 | No | 102.2 ± 5.2 | No |
| 67 | D 4476 | CK1 | 95.9 ± 8.8 | No | 93.1 ± 7.3 | No |
| 68 | EO 1428 | p38 MAPK | 45.3 ± 8.8 | Yes | 53.7 ± 8.1 | Yes |
| 69 | H 89 dihydrochloride | PKA | 102 ± 5.3 | No | 107.5 ± 3.1 | No |
| 70 | FPA 124 | PKB | Toxic | NA | Toxic | NA |
| 71 | GW 843682X | PLK | 97 ± 3.4 | No | 99.4 ± 2 | No |
| 72 | Iressa | EGFR | 106.1 ± 2 | No | 108.3 ± 11.7 | No |
| 73 | SU 5416 | VEGFR | 103.4 ± 1.7 | No | 92.1 ± 7.5 | No |
| 74 | 1-Naphthyl PP1 | Src | 98.2 ± 5.5 | No | 89.6 ± 13.6 | No |
| 75 | Dorsomorphin dihydrochloride | AMPK | Toxic | NA | Toxic | NA |
| 76 | BIO | GSK-3 | 43.2 ± 3.7 | Yes | 57.8 ± 7.7 | Yes |
| 77 | SD 208 | TGFbR1 | 100 ± 3.2 | No | 100 ± 2.3 | No |
| 78 | Compound 401 | DNA-PK | 103.4 ± 4.4 | No | 105.5 ± 8.9 | No |
| 79 | BI 78D3 | JNK | 1.9 ± 1.2 | Yes | 97.5 ± 7.2 | No |
| 80 | SC 514 | IKK | Toxic | NA | Toxic | NA |

^#^ 1 μM inhibitor (all others were 10 μM). * cMET inhibitor PHA 665752 induced large accumulation of fluorescent lysosomal-like structures precluding analysis of stress granule formation.

Toxic: some inhibitors were toxic to cells even at 1 μM, precluding stress granule assessment.

Underlined indicates decreased TDP-43 positive stress granules with no decrease in HuR-positive stress granules.
