# Supplementary material for: Kinase Inhibitor Screening Identifies Cyclin-Dependent Kinases and Glycogen Synthase Kinase 3 as Potential Modulators of TDP-43 Cytosolic Accumulation during Cell Stress
Source: PLoS One. 2013 Jun 26;8(6):e67433. doi: 10.1371/journal.pone.0067433 (PMC3694067; doi:10.1371/journal.pone.0067433)
Supplement: Table S3 — List of kinase classes associated with formation of TDP-43 and/or HuR-positive stress granules induced by paraquat treatment in SH-SY5Y cells. (DOCX) [file pone.0067433.s013.docx]

**Table S3:** List of kinase classes associated with formation of TDP-43 and/or HuR-positive stress granules induced by paraquat treatment in SH-SY5Y cells.

| **Target kinase** | **Kinase inhibitor number** | **Kinase inhibitor name** | **Inhibition of TDP-43 stress granules (from Table S2)** | **Inhibition of HuR stress granules (from Table S2)** |
| --- | --- | --- | --- | --- |
| p38 MAPK | 11 | SB 202190 | No | No |
| p38 MAPK | 19 | SB 203580 hydrochloride | Yes | Yes |
| p38 MAPK | 32 | SB 239063 | Yes | Yes |
| p38 MAPK | 68 | EO 1428 | Yes | Yes |
| CDK | 12 | Olomoucine | Yes | No |
| CDK | 24 | Purvalanol A | No | No |
| CDK | 25 | Purvalanol B | Yes | Yes |
| CDK | 31 | NSC 693868 | No | No |
| CDK | 35 | Aminopurvalanol A | Yes | Yes |
| CDK | 45 | Arcyriaflavin A | Yes | No |
| CDK | 57 | Ryuvidine | Yes | No |
| Aurora/CDK | 46 | ZM 447439 | Yes | No |
| Chk1 | 54 | SB 218078 | No | No |
| CK2 | 40 | TBB | Yes | No |
| EGFR | 1 | AG 490 | No | No |
| EGFR | 3 | AG 213 | No | No |
| EGFR | 6 | Genistein | Yes | No |
| EGFR | 38 | GW 583340 dihydrochloride | No | No |
| EGFR | 43 | BIBX 1382 dihydrochloride | Yes | No |
| EGFR | 72 | Iressa | No | No |
| GSK-3 | 28 | SB 216763 | Yes | No |
| GSK-3 | 29 | SB 415286 | Yes | No |
| GSK-3 | 76 | BIO | Yes | Yes |
| JAK3 | 15 | ZM 449829 | No | No |
| JAK3 | 16 | ZM 39923 hydrochloride | No | No |
| JAK2 | 41 | 1,2,3,4,5,6-Hexabromocyclohexane | No | No |
| JNK | 23 | SP 600125 | Yes | No |
| JNK | 79 | BI 78D3 | Yes | No |
| MEK | 8 | U0126 | Yes | Yes |
| MEK | 9 | PD 98059 | Yes | Yes |
| MEK | 30 | Arctigenin | Yes | Yes |
| MEK | 33 | SL 327 | Yes | Yes |
| MEK | 56 | PD 198306 | No | No |
| PI3K | 7 | LY 294002 hydrochloride | Yes | No |
| PI3K | 65 | PI 828 | No | No |
| PKB | 52 | 10-DEBC hydrochloride | No | Yes |
| PKC | 5 | GF 109203X | No | No |
| PKC | 26 | Rottlerin | No | No |
| PKC | 44 | CGP 53353 | Yes | No |
| Raf | 14 | ZM 336372 | Yes | Yes |
| Raf | 17 | GW 5074 | Yes | Yes |
| ROCK | 4 | Fasudil hydrochloride | No | No |
| ROCK | 10 | Y-27632 dihydrochloride | No | No |
| ROCK | 42 | HA 1100 hydrochloride | Yes | No |

Underlined indicates decreased TDP-43 positive stress granules with no decrease in HuR-positive stress granules.
