# Supplementary material for: Kinase Inhibitor Screening Identifies Cyclin-Dependent Kinases and Glycogen Synthase Kinase 3 as Potential Modulators of TDP-43 Cytosolic Accumulation during Cell Stress
Source: PLoS One. 2013 Jun 26;8(6):e67433. doi: 10.1371/journal.pone.0067433 (PMC3694067; doi:10.1371/journal.pone.0067433)
Supplement: Table S4 — Dose-response effect of representative kinase inhibitors on TDP-43-positive stress granules and inhibition of target kinase phosphorylation. (DOCX) [file pone.0067433.s014.docx]

**Table S4:** Dose-response effect of representative kinase inhibitors on TDP-43-positive stress granules and inhibition of target kinase phosphorylation.

| **Kinase inhibitor number** | **Kinase inhibitor name** | **Kinase inhibitor concentration (μM)** | **Target kinase** | **TDP-43 stress granule-positive cells**  **(% of paraquat treated cells)** | **Inhibition of target kinase**  **(% of paraquat treated cells)** |
| --- | --- | --- | --- | --- | --- |
| **0** | **Paraquat only** | **-** | **-** | **100 ± 7** | **-** |
| 8 | U0126 | 10  1  0.1 | MEK | 14 ± 6  18 ± 6  39 ± 8 | 9 ± 6  27 ± 11  56 ± 8 |
| 12 | Olomoucine | 10  1  0.1 | CDK | 4 ± 2  44 ± 16  103 ± 3 | 14 ± 8  62 ± 3  94 ± 5 |
| 19 | SB 203580 | 10  1  0.1 | p38 | 71 ± 11  84 ± 7  93 ± 9 | 26 ± 13  31 ± 9  67 ± 12 |
