# Supplementary material for: Kinase Inhibitor Screening Identifies Cyclin-Dependent Kinases and Glycogen Synthase Kinase 3 as Potential Modulators of TDP-43 Cytosolic Accumulation during Cell Stress
Source: PLoS One. 2013 Jun 26;8(6):e67433. doi: 10.1371/journal.pone.0067433 (PMC3694067; doi:10.1371/journal.pone.0067433)
Supplement: Table S5 — Effect of kinase inhibitors on TDP-43, hnRNP K, TIAR and HuR-positive stress granule formation induced by paraquat treatment in SH-SY5Y cells. (DOCX) [file pone.0067433.s015.docx]

**Table S5:** Effect of kinase inhibitors on TDP-43, hnRNP K, TIAR and HuR-positive stress granule formation induced by paraquat treatment in SH-SY5Y cells.

| **Kinase inhibitor number** | **Kinase inhibitor name** | **Target kinase** | **TDP-43 stress granule-positive cells**  **(% of paraquat treated cells)^1^** | **hnRNP K stress granule-positive cells**  **(% of paraquat treated cells)** | **TIAR stress granule-positive cells**  **(% of paraquat treated cells)** | **HuR stress granule-positive cells**  **(% of paraquat treated cells)^1^** |  |
| --- | --- | --- | --- | --- | --- | --- | --- |
| **0** | **Paraquat only** | **-** | **100 ± 4.4** | **100 ± 4.2** | **100 ± 4.8** | **100 ± 2.6** |  |
| 5 | GF 109203X | PKC | 73.8 ± 6.3 | 85.7 ± 6.6 | 89.1 ± 3 | 78.4 ± 3.2 |  |
| 7 | LY 294002 hydrochloride | PI3K | 36.8 ± 5 | 40 ± 4 | 25.9 ± 2.4 | 97.7 ± 3.4 | |
| 8 | U0126 | MEK | 12.3 ± 4.7 | 14 ± 6.8 | nd | 27.2 ± 8.5 |  |
| 9 | PD 98059 | MEK | 18 ± 2.9 | 23.7 ± 5.6 | 18.9 ± 2 | 14.6 = 6.3 |  |
| 11 | SB 202190 | p38 MAPK | 152.5 ± 5.6 | 161.1 ± 10.3 | nd | 105 ± 3.3 |  |
| 12 | Olomoucine | CDK | 6.6 ± 2.8 | 7.1 ± 2.1 | 11.5 ± 4.6 | 98.2 ± 2.4 |  |
| 17 | GW 5074 | Raf | 19.2 ± 2.6 | 8.4 ± 6.5 | 26.9 ± 3.7 | 5.4 ± 0.6 |  |
| 19 | SB 203580 hydrochloride | p38 MAPK | 62.9 ± 13.7 | 78 ± 12.4 | 58.1 ± 5.5 | 55.1 ± 11.6 |  |
| 23 | SP 600125 | JNK | 2.4 ± 1.7 | 3.6 ± 1.7 | 1.7 ± 1.1 | 97.2 ± 6.6 |  |
| 29^#^ | SB 415286 | GSK-3 | 9 ± 4.4 | 14.9 ± 3.7 | 11.8 ± 5.7 | 97 ± 2 |  |
| 30 | Arctigenin | MEK | 42.7 ± 4.3 | 20 ± 2.4 | 20.3 ± 2.8 | 43.3 ± 1.7 |  |
| 32^#^ | SB 239063 | p38 MAPK | 13.9 ± 3.7 | 18.7 ± 9.3 | nd | 18.2 ± 6.3 |  |
| 35^#^ | Aminopurvalanol A | CDK | 2.8 ± 1.7 | 27.2 ± 2.4 | 15.4 ± 3 | 34 ± 1.8 |  |
| 42 | HA 1100 hydrochloride | ROCK | 15.3 ± 7.3 | 24.8 ± 8 | nd | 118.2 ± 12.8 |  |
| 44 | CGP 53353 | PKC | 33 ± 3 | 27.9 ± 1.3 | 14.9 ± 1.6 | 100 ± 2 | |
| 45 | Arcyriaflavin A | CDK | 2.7 ± 1.5 | 3.6 ± 3.1 | 14 ± 7.3 | 126.3 ± 14.9 |  |
| 46 | ZM 447439 | Aurora | 55.5 ± 11.2 | 48.5 ± 5.7 | 59.3 ± 3.2 | 107.2 ± 12 |  |

^#^ 1 μM inhibitor (all others were 10 μM). nd = not done. ^1^ = Data from Table S2 for TDP-43 and HuR is included for comparison with hnRNP K and TIAR.
