# Supplementary material for: Kinase Inhibitor Screening Identifies Cyclin-Dependent Kinases and Glycogen Synthase Kinase 3 as Potential Modulators of TDP-43 Cytosolic Accumulation during Cell Stress
Source: PLoS One. 2013 Jun 26;8(6):e67433. doi: 10.1371/journal.pone.0067433 (PMC3694067; doi:10.1371/journal.pone.0067433)
Supplement: Table S6 — Comparison of kinases associated with formation of TDP-43, hnRNP K, TIAR and/or HuR-positive stress granules induced by paraquat treatment in SH-SY5Y cells. (DOCX) [file pone.0067433.s016.docx]

**Table S6:** Comparison of kinases associated with formation of TDP-43, hnRNP K, TIAR and/or HuR-positive stress granules induced by paraquat treatment in SH-SY5Y cells.

| **Target kinase** | **Kinase inhibitor number** | **Kinase inhibitor name** | **Inhibition of TDP-43 stress granules (P<0.05 compared to control, Yes/No)^1^** | **Inhibition of hnRNP K stress granules (P<0.05 compared to control, Yes/No)** | **Inhibition of TIAR stress granules (P<0.05 compared to control, Yes/No)** | **Inhibition of HuR stress granules (P<0.05 compared to control, Yes/No)^1^** |
| --- | --- | --- | --- | --- | --- | --- |
| p38 MAPK | 11 | SB 202190 | No | No | nd | No |
| p38 MAPK | 19 | SB 203580 hydrochloride | Yes | No | Yes | Yes |
| p38 MAPK | 32 | SB 239063 | Yes | Yes | nd | Yes |
| CDK | 12 | Olomoucine | Yes | Yes | Yes | No |
| CDK | 35 | Aminopurvalanol A | Yes | Yes | Yes | Yes |
| CDK | 45 | Arcyriaflavin A | Yes | Yes | Yes | No |
| Aurora/CDK | 46 | ZM 447439 | Yes | Yes | Yes | No |
| GSK-3 | 29 | SB 415286 | Yes | Yes | Yes | No |
| JNK | 23 | SP 600125 | Yes | Yes | Yes | No |
| MEK | 8 | U0126 | Yes | Yes | nd | Yes |
| MEK | 9 | PD 98059 | Yes | Yes | Yes | Yes |
| MEK | 30 | Arctigenin | Yes | Yes | Yes | Yes |
| PI3K | 7 | LY 294002 hydrochloride | Yes | Yes | Yes | No |
| PKC | 5 | GF 109203X | No | No | No | No |
| PKC | 44 | CGP 53353 | Yes | Yes | Yes | No |
| Raf | 17 | GW 5074 | Yes | Yes | Yes | Yes |
| ROCK | 42 | HA 1100 hydrochloride | Yes | Yes | nd | No |

nd = not done.

^1^ = Data from Table S3 for TDP-43 and HuR is included for comparison with hnRNP K and TIAR.
