# Supplementary material for: Kinase Inhibitor Screening Identifies Cyclin-Dependent Kinases and Glycogen Synthase Kinase 3 as Potential Modulators of TDP-43 Cytosolic Accumulation during Cell Stress
Source: PLoS One. 2013 Jun 26;8(6):e67433. doi: 10.1371/journal.pone.0067433 (PMC3694067; doi:10.1371/journal.pone.0067433)
Supplement: Table S7 — Effect of kinase inhibitors on formation of TDP-43-positive stress granules induced by paraquat treatment in retinoic-acid treated compared to non-treated SH-SY5Y cells. (DOCX) [file pone.0067433.s017.docx]

**Table S7:** Effect of kinase inhibitors on formation of TDP-43-positive stress granules induced by paraquat treatment in retinoic-acid treated compared to non-treated SH-SY5Y cells.

| **Kinase inhibitor number** | **Kinase inhibitor name** | **Target kinase** | **TDP-43 stress granule-positive cells in retinoic acid-treated cultures.**  **(% of paraquat treated cells)** | **TDP-43 stress granule-positive cells in non-retinoic acid- treated cultures.**  **(% of paraquat treated cells)^1^** | | **Concordance between retinoic and non-retinoic acid-treated cells.** |
| --- | --- | --- | --- | --- | --- | --- |
| **0** | **Paraquat only** | **-** | **100 ± 5.9** | **100 ± 4.4** | **na** | |
| 5 | GF 109203X | PKC | 95.3 ± 3.7 | 73.8 ± 6.3 | Yes | |
| 7 | LY 294002 hydrochloride | PI3K | 47.4 ± 7.8* | 38.6 ± 5* | Yes | |
| 8 | U0126 | MEK | 23.9 ± 5.7* | 12.3 ± 4.7* | Yes | |
| 9 | PD 98059 | MEK | 9.5 ± 8.7* | 18 ± 2.9* | Yes | |
| 11 | SB 202190 | p38 MAPK | 108.8 ± 9.4 | 152.5 ± 5.6 | No | |
| 12 | Olomoucine | CDK | 14.2 ± 6.1* | 6.6 ± 2.8* | Yes | |
| 17 | GW 5074 | Raf | 26.1 ± 7* | 19.2 ± 2.6* | Yes | |
| 19 | SB 203580 hydrochloride | p38 MAPK | 47.3 ± 6.6* | 62.9 ± 13.7* | Yes | |
| 23 | SP 600125 | JNK | 3.9 ± 2* | 2.4 ± 1.7* | Yes | |
| 29^#^ | SB 415286 | GSK-3 | 17.3 ± 7.2* | 9 ± 4.4* | Yes | |
| 30 | Arctigenin | MEK | 56.2 ± 3.2* | 42.7 ± 4.3* | Yes | |
| 32^#^ | SB 239063 | p38 MAPK | 28.5 ± 6.7* | 13.9 ± 3.7* | Yes | |
| 35^#^ | Aminopurvalanol A | CDK | 4.5 ± 3.1* | 2.8 ± 1.7* | Yes | |
| 42 | HA 1100 hydrochloride | ROCK | 8.8 ± 3.2* | 15.3 ± 7.3* | Yes | |
| 44 | CGP 53353 | PKC | 38.8 ± 6.4* | 33 ± 3* | Yes | |
| 45 | Arcyriaflavin A | CDK | 1.3 ± 1.1* | 2.7 ± 1.5* | Yes | |
| 46 | ZM 447439 | Aurora | 104.3 ± 8.9 | 55.5 ± 11.2* | No | |

^#^ 1 μM inhibitor (all others were 10 μM). *P<0.05, inhibition compared to paraquat only.

^1^ = Data from Table S2 for TDP-43 is included for comparison with retinoic acid-treated cells.
