# Supplementary material for: Kinase Inhibitor Screening Identifies Cyclin-Dependent Kinases and Glycogen Synthase Kinase 3 as Potential Modulators of TDP-43 Cytosolic Accumulation during Cell Stress
Source: PLoS One. 2013 Jun 26;8(6):e67433. doi: 10.1371/journal.pone.0067433 (PMC3694067; doi:10.1371/journal.pone.0067433)
Supplement: Table S8 — Effect of kinase inhibitors on formation of TDP-43 and HuR-positive stress granules induced by sodium arsenite treatment in HeLa epithelial cells. (DOCX) [file pone.0067433.s018.docx]

**Table S8:** Effect of kinase inhibitors on formation of TDP-43 and HuR-positive stress granules induced by sodium arsenite treatment in HeLa epithelial cells.

| **Kinase inhibitor number** | **Kinase inhibitor name** | **Target kinase** | **TDP-43 stress granules**  **(% of paraquat treated cells)** | **HuR stress granules**  **(% of paraquat treated cells)** |
| --- | --- | --- | --- | --- |
| **0** | **Sodium arsenite only** | **-** | **100 ± 6.7** | **100 ± 9.2** |
| 12 | Olomoucine | CDK | 1.2 ± 0.9* | 107.3 ± 11.2 |
| 17 | GW 5074 | Raf | 67.3 ± 12.1* | 47.3 ± 3.7* |
| 19 | SB 203580 hydrochloride | p38 MAPK | 43.2 ± 8.3* | 61.2 ± 4* |
| 30 | Arctigenin | MEK | 4.1 ± 1.2* | 8.3 ± 4.8* |
| 32^#^ | SB 239063 | p38 MAPK | 0.3 ± 0.1* | 2.7 ± 1.7* |
| 35^#^ | Aminopurvalanol A | CDK | 3.6 ± 3.1* | 103 ± 7 |
| 45 | Arcyriaflavin A | CDK | 2.9 ± 1.7* | 72.3 ± 6.9 |

^#^ 1 μM inhibitor (all others were 10 μM).

*P<0.05, inhibition compared to sodium arsenite alone.
