# Supplementary material for: Kinase Inhibitor Screening Identifies Cyclin-Dependent Kinases and Glycogen Synthase Kinase 3 as Potential Modulators of TDP-43 Cytosolic Accumulation during Cell Stress
Source: PLoS One. 2013 Jun 26;8(6):e67433. doi: 10.1371/journal.pone.0067433 (PMC3694067; doi:10.1371/journal.pone.0067433)
Supplement: Table S9 — Ability of kinase inhibitors to reverse TDP-43 and/or HuR-positive stress granule formation induced by paraquat treatment when for the final 6 hr of 24 hr paraquat treatment (SH-SY5Y cells). (DOCX) [file pone.0067433.s019.docx]

**Table S9:** Ability of kinase inhibitors to reverse TDP-43 and/or HuR-positive stress granule formation induced by paraquat treatment when for the final 6 hr of 24 hr paraquat treatment (SH-SY5Y cells).

| **Kinase inhibitor number** | **Kinase inhibitor name** | **Target kinase** | **TDP-43 stress granule-positive cells**  **(% of paraquat treated cells)** | **HuR stress granule-positive cells**  **(% of paraquat treated cells)** |
| --- | --- | --- | --- | --- |
| **0** | **Paraquat only** | **-** | **100 ± 3.1** | **100 ± 2.2** |
| 7 | LY 294002 hydrochloride | PI3K | 84.2 ± 4.2 | 94 ± 5 |
| 12 | Olomoucine | CDK | 2.1 ± 1.2* | 46.9 ± 5.3* |
| 19 | SB 203580 hydrochloride | p38 MAPK | 86 ± 8.5 | 96 ± 1.6 |
| 23 | SP 600125 | JNK | 90.1 ± 4.3 | 96 ± 1.9 |
| 29^#^ | SB 415286 | GSK-3 | 89.9 ± 2.2 | 99.6 ± 0.6 |
| 30 | Arctigenin | MEK | 100.2 ± 3.9 | 90.6 ± 2.7 |
| 32^#^ | SB 239063 | p38 MAPK | 97.5 ± 8.3 | 101.4 ± 5.6 |
| 35^#^ | Aminopurvalanol A | CDK | 1 ± 0.6* | 42.4 ± 2.4* |
| 42 | HA 1100 hydrochloride | ROCK | 104.7 ± 7.8 | 109.2 ± 4.3 |
| 45 | Arcyriaflavin A | CDK | 2 ± 1.1* | 89.9 ± 4.1 |
| 46 | ZM 447439 | Aurora/CDK | 47.3 ± 6.7* | 75.5 ± 7.4* |

^#^ 1 μM inhibitor (all others were 10 μM).

*P<0.05, inhibition compared to paraquat only.
